# Common elective orthopaedic procedures and their clinical effectiveness: umbrella review of level 1 evidence

**DOI:** 10.1136/bmj.n1511

**Published:** 2021-07-08

**Authors:** Ashley W Blom, Richard L Donovan, Andrew D Beswick, Michael R Whitehouse, Setor K Kunutsor

**Affiliations:** 1 National Institute for Health Research Bristol Biomedical Research Centre, University Hospitals Bristol and Weston NHS Foundation Trust and the University of Bristol, Bristol, UK; 2 Musculoskeletal Research Unit, Translational Health Sciences, Bristol Medical School, University of Bristol, Learning & Research Building (Level 1), Southmead Hospital, Bristol, UK

## Abstract

**Objective:**

To determine the clinical effectiveness of common elective orthopaedic procedures compared with no treatment, placebo, or non-operative care and assess the impact on clinical guidelines.

**Design:**

Umbrella review of meta-analyses of randomised controlled trials or other study designs in the absence of meta-analyses of randomised controlled trials.

**Data sources:**

Ten of the most common elective orthopaedic procedures—arthroscopic anterior cruciate ligament reconstruction, arthroscopic meniscal repair of the knee, arthroscopic partial meniscectomy of the knee, arthroscopic rotator cuff repair, arthroscopic subacromial decompression, carpal tunnel decompression, lumbar spine decompression, lumbar spine fusion, total hip replacement, and total knee replacement—were studied. Medline, Embase, Cochrane Library, and bibliographies were searched until September 2020.

**Eligibility criteria for selecting studies:**

Meta-analyses of randomised controlled trials (or in the absence of meta-analysis other study designs) that compared the clinical effectiveness of any of the 10 orthopaedic procedures with no treatment, placebo, or non-operative care.

**Data extraction and synthesis:**

Summary data were extracted by two independent investigators, and a consensus was reached with the involvement of a third. The methodological quality of each meta-analysis was assessed using the Assessment of Multiple Systematic Reviews instrument. The Jadad decision algorithm was used to ascertain which meta-analysis represented the best evidence. The National Institute for Health and Care Excellence Evidence search was used to check whether recommendations for each procedure reflected the body of evidence.

**Main outcome measures:**

Quality and quantity of evidence behind common elective orthopaedic interventions and comparisons with the strength of recommendations in relevant national clinical guidelines.

**Results:**

Randomised controlled trial evidence supports the superiority of carpal tunnel decompression and total knee replacement over non-operative care. No randomised controlled trials specifically compared total hip replacement or meniscal repair with non-operative care. Trial evidence for the other six procedures showed no benefit over non-operative care.

**Conclusions:**

Although they may be effective overall or in certain subgroups, no strong, high quality evidence base shows that many commonly performed elective orthopaedic procedures are more effective than non-operative alternatives. Despite the lack of strong evidence, some of these procedures are still recommended by national guidelines in certain situations.

**Systematic review registration:**

PROSPERO CRD42018115917.

## Introduction

About 200 musculoskeletal conditions have a substantial effect on the quality of life of millions of people in the UK and are associated with increased healthcare costs.^1^ According to the World Health Organization, the most common and disabling musculoskeletal conditions are osteoarthritis, back and neck pain, fractures associated with fragility of the bone, injuries, and systemic inflammatory conditions such as rheumatoid arthritis. Musculoskeletal conditions are typically characterised by persistent pain and restricted mobility. According to the 2017 Global Burden of Disease study, musculoskeletal conditions were the biggest contributor to global disability.^2^ Although many musculoskeletal conditions can be managed in primary care through a combination of core interventions such as exercise, weight management, physical therapies, psychological therapies, and drug treatment, some patients who do not respond to conservative measures need specialist care, surgical care, or both.^3^ In the UK, musculoskeletal conditions account for more than 25% of all surgical interventions in the National Health Service (NHS).

Total joint replacement is one of the most common elective orthopaedic procedures performed worldwide for end stage osteoarthritis, the most common musculoskeletal condition.^4^ Although total joint replacement is considered to be a clinically effective intervention for the management of osteoarthritis, it also accounts for enormous expenditures in the health system. The term “clinical effectiveness” is about ensuring that healthcare practice is based on the best available data and evidence of effectiveness. The National Institute for Health and Care Excellence (NICE) defines the “clinical effectiveness of a treatment” as how beneficial the treatment is under usual or everyday conditions, compared with doing nothing or opting for another type of care.^5^ Randomised controlled trials are considered the best method to evaluate the clinical effectiveness of an intervention. They deliver the highest level of evidence owing to their potential to minimise bias. Concerns have been expressed that many orthopaedic surgical interventions, as well as prostheses used in these interventions, do not have readily available or high quality evidence on their clinical effectiveness to support their use.^6^ ^7^ A recent review found that 24% of all hip replacement implants available to surgeons in the UK did not have evidence for their clinical effectiveness.^8^ Although clinical guidelines, defined as “systematically developed statements to assist practitioner and patient decisions about appropriate health care for specific clinical circumstances,”^9^ aim to be informed by the best available evidence, they have often been criticised for their lack of methodological rigour and applicability.^10^ ^11^ A critical appraisal of existing treatment guidelines for the management of hip and knee osteoarthritis by the Osteoarthritis Research Society International’s Treatment Guidelines Committee observed that the overall quality of existing guidelines was suboptimal and consensus recommendations were not always supported by the best available evidence.

Whether common orthopaedic procedures for managing musculoskeletal conditions are accompanied by high quality evidence bases is unclear. In this context, using an umbrella review of systematic reviews/meta-analyses of randomised controlled trials or other study designs in the absence of meta-analyses of randomised controlled trials, we aimed to evaluate the clinical effectiveness of the 10 most common elective orthopaedic procedures, assess whether the evidence base on these 10 orthopaedic procedures has affected guidelines, and identify gaps in the existing evidence.

## Methods

### Protocol and registration

We did an umbrella review—a systematic collection and critical evaluation of multiple systematic reviews/meta-analyses on a specific research topic.^12^ This was conducted according to PRISMA (Preferred Reporting Items for Systematic Reviews and Meta-​Analyses) and MOOSE (Meta-analyses of Observational Studies in Epidemiology) guidelines^13^ ^14^ (appendices 1 and 2) and was based on a predefined protocol registered in the PROSPERO international prospective register of systematic reviews (CRD42018115917).

### Selection of 10 common elective orthopaedic procedures

To identify the 10 most elective common orthopaedic procedures and their indications (table 1), we combined a literature search for relevant articles on the topic, an assessment of Hospital Episode Statistics procedure frequency counts, and discussions with prominent and experienced orthopaedic surgeons. We used these three approaches in parallel and compared their results. As the results were generally concordant, no further selection or arbitration process was needed.

**Table 1:** Common elective orthopaedic procedures and indications

Procedure	Main indication
Arthroscopic anterior cruciate ligament reconstruction	Anterior cruciate ligament rupture
Arthroscopic meniscal repair of the knee	Traumatic meniscal tears
Arthroscopic partial meniscectomy of the knee	Degenerative meniscal tears
Arthroscopic rotator cuff repair	Acute rotator cuff tears
Arthroscopic subacromial decompression	Subacromial impingement syndrome
Carpal tunnel decompression	Carpal tunnel syndrome
Lumbar spine decompression	Spinal canal stenosis
Lumbar spine fusion	Degenerative disc disease
Total hip replacement	End stage osteoarthritis
Total knee replacement	End stage osteoarthritis

### Data sources, search strategy, and study selection

We searched Medline, Embase, and the Cochrane Library from inception to September 2020 for studies that compared the clinical effectiveness of each of the selected orthopaedic interventions with no treatment, placebo, or non-operative care. We did separate searches for each orthopaedic procedure. The computer based searches combined free and MeSH search terms and keywords related to the orthopaedic procedure (for example, “total knee replacement”, “total hip replacement”, “carpal tunnel surgery”) and population (for example, “osteoarthritis”, “carpal tunnel syndrome”, “subacromial impingement syndrome”). We applied filters for systematic reviews/meta-analyses. We restricted the search to articles written in the English language because our study design was an umbrella review of systematic reviews/meta-analyses and we took into consideration the extent of the literature included by each contributing review during our selection of the best currently available evidence for a procedure. The detailed strategy for each orthopaedic procedure is reported in appendix 3. We screened all titles and abstracts of retrieved citations to assess suitability for inclusion. Two authors (SKK and RLD) independently evaluated the full text of articles potentially meeting eligibility criteria for study selection. When necessary, discrepancies were discussed and consensus reached with the involvement of a third author (MRW). To account for studies missed in the original search, we manually scanned reference lists of eligible articles.

### Eligibility criteria

Studies were eligible if they were systematic reviews/meta-analyses including randomised controlled trials that compared the clinical effectiveness of any of the orthopaedic procedures mentioned above with no treatment, placebo, or non-operative care. In the absence of meta-analyses of randomised controlled trials, we sought individual randomised controlled trials followed by systematic reviews/meta-analyses of observational cohort studies, in that order, according to the hierarchy of evidence (appendix 4).^15^ We excluded network meta-analyses (when pairwise meta-analyses were available), narrative reviews, systematic reviews that did not pool data or do a meta-analysis, and meeting abstracts. For multiple Cochrane reviews evaluating the same topic, we selected the most recently updated one.

### Data extraction, quality, and risk of bias assessment

One author (SKK) initially extracted data by using a standardised data collection form. A second author (RLD) independently checked the extracted data against the original articles. In the event of discrepancies between authors, a third author (MRW) was consulted. We extracted information on the first author’s name, year and journal of publication, databases searched, number of component randomised controlled trials, outcomes and findings, type of effect model used in the meta-analysis (fixed or random), between study heterogeneity estimates (I^2^ values), any presented measure of publication bias, Grading of Recommendations Assessment, Development and Evaluation (GRADE) rating, sources of funding, and conflicts of interest. We explored whether the studies evaluated possible sources of heterogeneity across studies by using methods such as subgroup analyses and whether the authors formally conducted a sensitivity analysis.

We assessed the methodological quality of each included meta-analysis by using the Assessment of Multiple Systematic Reviews (AMSTAR) instrument,^16^ an 11 item tool that is widely recognised for assessing the methodological quality of systematic reviews/meta-analyses and has good reliability and external validity.^17^ ^18^ The tool includes ratings for quality in the search, analysis, and transparency of a meta-analysis. The maximum score achievable by a review is 11. Regarding the rating item for methodological quality in the analysis, we downgraded any study that had used a fixed effects model rather than a random effects model for producing a summary estimate. We considered the random effects model to be the most appropriate pooling approach given the heterogeneity in study designs and populations in orthopaedic research.

### Selecting best body of evidence for each procedure

**Fig 1:**
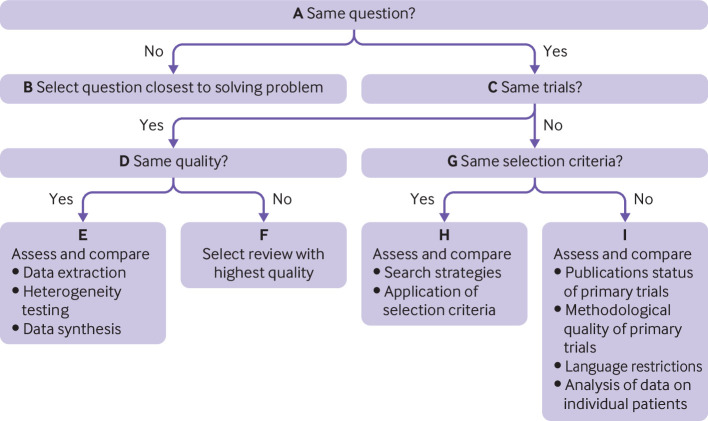
Jadad decision algorithm

We used the Jadad decision algorithm (fig 1) to provide recommendations for the use of each orthopaedic procedure.^19^ Given the conflicts among systematic reviews/meta-analyses that produce difficulties for decision makers in making recommendations on a particular intervention, the Jadad decision algorithm was developed to help decision makers to select among discordant reviews.^19^ This tool determines the source of discordance between systematic reviews, including differences in clinical question, inclusion and exclusion criteria, data extraction, quality assessment, data pooling, and statistical analysis (fig 1). It has been and is now widely used to develop treatment recommendations from among meta-analyses with discordant results.^20^ ^21^ Two authors independently applied the algorithm and arrived at a consensus as to which of the meta-analyses provided the best available evidence. We did not use the Jadad decision algorithm if all meta-analyses for an orthopaedic procedure considered the same study question and reported similar results. Furthermore, given that randomised controlled trials included in each meta-analysis varied in their methodological quality, we needed to put the overall findings in the context of the best available evidence (as a type of sensitivity analysis). Hence, we selected and summarised the results of randomised controlled trials with a low risk of bias from each meta-analysis, representing the best body of evidence for each procedure. Finally, following the collection of the evidence, we compared findings for each procedure with national guidelines (NICE evidence search) to check whether recommendations for each procedure reflected the body of available evidence.

### Patient and public involvement

In the Musculoskeletal Research Unit of the University of Bristol and North Bristol NHS Trust, we have a dedicated patient and public involvement group, the Patient Experience Partnership in Research (PEP-R).^22^ This comprises patients, all of whom have undergone core treatments for various musculoskeletal conditions. It is a facilitated group that works in partnership with researchers to provide patient and public input into research and helps to identify avenues for the dissemination of research findings. Members of the research team have regularly met with this group to ensure that every research project in the unit remains relevant to patients. Our regular meetings with the group inspired this review.

## Results

**Fig 2:**
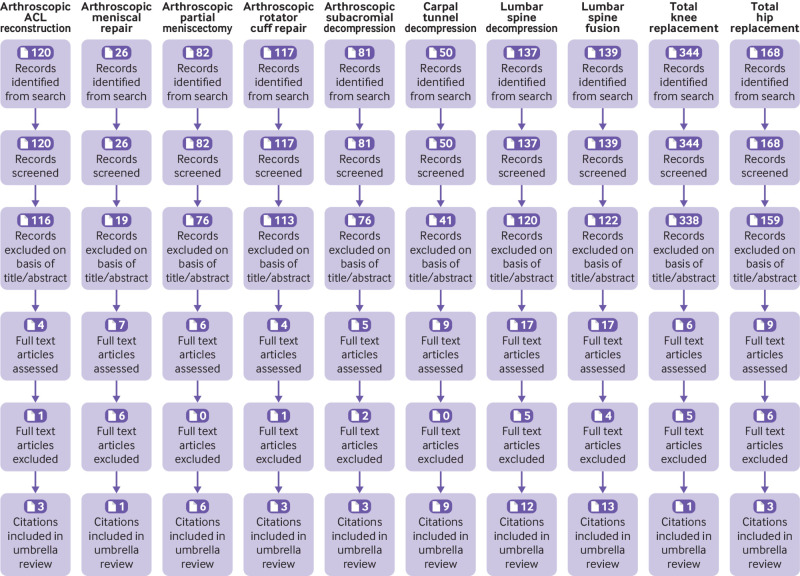
PRISMA flow diagram. ACL=anterior cruciate ligament

The study selection process from retrieval of search results to inclusion in the umbrella review is illustrated in the PRISMA flow diagram (fig 2), according to each orthopaedic procedure. A full reference list of the included studies is provided in appendix 5.

### Arthroscopic anterior cruciate ligament reconstruction for anterior cruciate ligament deficiency

**Fig 3:**
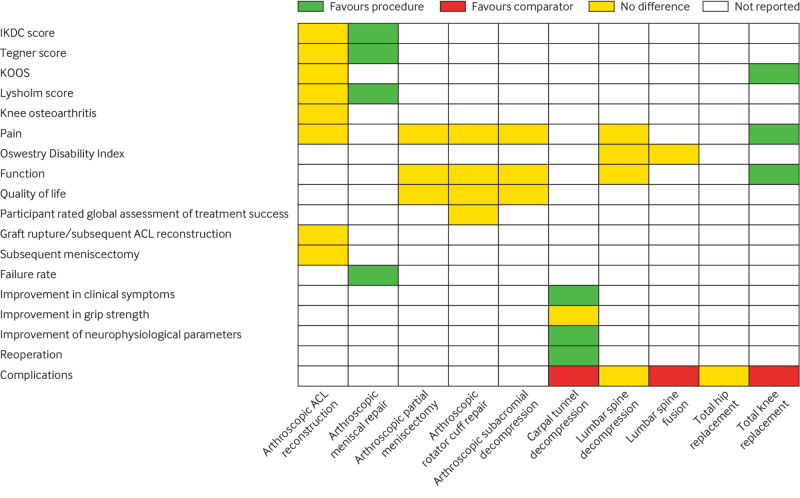
Overall findings of umbrella review by outcome measures and orthopaedic procedures. ACL=anterior cruciate ligament; IKDC=International Knee Documentation Committee; KOOS=Knee Injury and Osteoarthritis Outcome Score

Of 120 citations retrieved for anterior cruciate ligament reconstruction, three articles were eligible for the review (fig 2; appendix 5). Publication dates ranged from 2014 to 2020, and AMSTAR scores ranged from 9 to 11 (appendices 6 and 7). Two reviews included a mix of comparative observational cohort studies and one randomised controlled trial each, which compared surgical treatment (anterior cruciate ligament reconstruction) with non-surgical treatment. The third was a Cochrane review, which evaluated one randomised controlled trial. All three reviews in their pooled analyses showed no difference in patient reported outcome measures (fig 3), and the GRADE quality of the evidence was low. A total of two randomised controlled trials were included in the three reviews, and each reported no differences in patient reported outcome measures between surgical treatment and non-surgical treatment. The randomised controlled trial by Frobell and colleagues, which compared structured rehabilitation plus early anterior cruciate ligament reconstruction with structured rehabilitation plus optional later anterior cruciate ligament reconstruction in 121 adults with acute anterior cruciate ligament injuries, represented the best body of evidence for anterior cruciate ligament reconstruction.^23^ ^24^ Patient reported or radiographic outcomes and adverse events did not differ between the two interventions after two and five years (appendices 8 and 9).

### Arthroscopic meniscal repair of the knee for traumatic tears

Of 26 retrieved articles, no reviews of randomised controlled trials comparing arthroscopic meniscal repair with no treatment, placebo, or non-operative care were identified (fig 2; appendix 5). However, we identified a review of mostly observational studies (including one randomised controlled trial) that compared meniscal repair (open suture and arthroscopic inside-out procedures) with meniscectomy (arthroscopic partial or total meniscectomy) (appendices 8 and 9). In this review, meniscal repairs were reported to have better long term patient reported outcome measures, better activity levels, and lower failure rates than meniscectomy (fig 3). In the single randomised controlled trial that compared a variety of surgical procedures (arthroscopic repair, arthroscopic minimal resection and repair, or arthroscopic partial meniscectomy) with non-surgical treatment,^25^ clinical findings based on International Knee Documentation Committee protocols suggested that non-surgical treatment was unsatisfactory.

### Arthroscopic partial meniscectomy of knee for degenerative tears

Of 82 retrieved articles on arthroscopic partial meniscectomy, six were eligible for evaluation (fig 2; appendix 5). Characteristics and findings of each meta-analysis are provided in appendix 6. Publication dates ranged from 2014 to 2020. AMSTAR scores ranged from 8 to 11 (appendix 7). In all six reviews of randomised controlled trials, arthroscopic partial meniscectomy did not show clinically important benefit over conservative treatment for knee function and pain. In the most recent review, which was based on 10 randomised controlled trials, arthroscopic partial meniscectomy did not provide a clinically meaningful improvement in knee pain, function, or quality of life (fig 3). However, small benefits of arthroscopic partial meniscectomy were reported for patients without osteoarthritis. The authors indicated that surgical treatment should not be considered the first line intervention for patients with knee pain and meniscal tear. The GRADE quality of the evidence ranged from low to high. Two randomised controlled trials were identified to represent the best body of evidence for arthroscopic partial meniscectomy.^26^ ^27^ In a comparison of arthroscopic partial meniscectomy plus postoperative physical therapy with a standardised physical therapy regimen in patients with symptoms, a meniscal tear, and evidence of mild to moderate osteoarthritis on imaging, no significant differences were seen between the study groups in functional improvement and frequency of adverse events.^26^ In 140 middle aged patients with degenerative meniscal tears, arthroscopic partial meniscectomy provided no clinically relevant difference in Knee Injury and Osteoarthritis Outcome Score compared with a 12 week supervised exercise programme, and no serious adverse events occurred in either group (appendices 8 and 9).^27^

### Arthroscopic rotator cuff repair for acute rotator cuff tears

Of 117 retrieved articles on rotator cuff repair, three meta-analyses were eligible for the review (fig 2; appendix 5). Their general characteristics and outcomes are provided in appendix 6. Their publication dates ranged from 2015 to 2019. AMSTAR scores ranged from 7 to 11 (appendix 7). One study compared arthroscopic versus mini-open rotator cuff repair and reported no differences in surgery time, functional outcome score, visual analogue scale pain score, and range of motion between the two techniques. In a Cochrane review that mainly compared arthroscopic rotator cuff repair with or without subacromial decompression versus non-operative treatment (exercises with or without glucocorticoid injection) as well as other comparisons, arthroscopic rotator cuff repair provided little or no clinically important benefits for pain, function, quality of life, or participant rated global assessment of treatment success compared with non-operative treatment (fig 3). In the third review, which compared surgical repair versus conservative treatment or subacromial decompression alone for degenerative rotator cuff tears, surgical repair resulted in significantly improved outcomes (Constant-Murley score) compared with other strategies. Surgical repair comprised a variety of techniques including mini-open, open, or arthroscopic rotator cuff repair, and no subgroup analysis was done for arthroscopic versus open repair. None of the trials included in the reviews compared arthroscopic rotator cuff repair with no treatment or placebo. The GRADE quality of the evidence ranged from very low to moderate. Given that the three reviews did not investigate the same research question (compared different strategies), we selected the review with the question closest to ours on the basis of the Jadad decision algorithm (fig 1). The best available was the Cochrane review,^28^ which showed no clinically important benefits of arthroscopic rotator cuff repair over non-operative care (fig 3). In two selected randomised controlled trials that represented the best body of evidence and compared rotator cuff repair with physiotherapy, no clinically important differences were observed between the two interventions at short term and long term follow-up (appendices 8 and 9).^29^ ^30^ ^31^ ^32^

### Arthroscopic subacromial decompression for subacromial impingement syndrome

We retrieved 81 articles on arthroscopic subacromial decompression, and three fulfilled the eligibility criteria (fig 2; appendix 5). A general description of the characteristics of each meta-analysis is provided in appendix 6. All studies were published in 2019 and 2020. AMSTAR scores ranged from 9 to 11 (appendix 7). All three reviews compared subacromial decompression surgery for subacromial impingement syndrome/subacromial pain syndrome/rotator cuff disease with any other treatment (placebo, exercise therapy, physiotherapy, or no intervention). All three reviews indicated that subacromial decompression did not provide clinically important improvement in pain, function, or quality of life compared with other treatments (fig 3). Two meta-analyses compared open versus arthroscopic subacromial decompression and reported similar clinical outcomes, although arthroscopic subacromial decompression was associated with a quicker return to work and shorter length of stay in hospital.^33^ ^34^ We identified two randomised controlled trials to represent the best body of evidence for arthroscopic subacromial decompression. They both reported no clinically important differences in patient reported outcomes and adverse events between arthroscopic subacromial decompression and placebo surgery (appendices 8 and 9).^35^ ^36^

### Carpal tunnel decompression for carpal tunnel syndrome

Of 50 retrieved citations on carpal tunnel decompression, nine meta-analyses of randomised controlled trials were eligible for the review (fig 2; appendix 5). Publication dates ranged from 2008 to 2019, and AMSTAR scores ranged from 7 to 11 (appendices 6 and 7). Eight of the reviews compared endoscopic or limited incision with open or standard incision carpal tunnel decompression for the treatment of carpal tunnel syndrome. Only one meta-analysis compared surgical versus non-surgical treatment.

In the single meta-analysis that synthesised four randomised controlled trials comparing surgical versus non-surgical treatment (splinting or local corticosteroid injections), surgical treatment was shown to relieve symptoms significantly better than non-surgical treatment; however, surgical treatment was associated with more complications (fig 3). No randomised controlled trials compared carpal tunnel decompression with placebo or no treatment.

For the comparison between endoscopic versus open carpal tunnel decompression, the results were discordant across the eight reviews. However, general findings suggested that endoscopic and open release are about as effective as each other in relieving symptoms and improving functional status, although endoscopic release may have functional benefits over the open release with regards to return to work and improved grip strength. Some evidence suggested that endoscopic release increased the risk of nerve injury compared with open release. The GRADE quality of the evidence ranged from very low to low. On the basis of the fact that most reviews that investigated the same research question did not include the same trials and selection criteria were similar, the Jadad decision algorithm indicated that the best available evidence should be chosen according to search strategies and application of selection criteria (fig 1). Hence, we selected the Cochrane review.^37^ The findings, which were based on very low to low quality GRADE evidence, indicated that open and endoscopic releases for carpal tunnel syndrome are about as effective as each other in relieving symptoms and improving functional status, although endoscopic release may have a functionally significant benefit over open release for improvement in grip strength. In all three selected randomised controlled trials that represented the best body of evidence and compared carpal tunnel surgery (mostly open release) with non-surgical treatment (wrist splinting, steroid injection, or hand therapy), surgery was more effective in most outcome measures (overall symptom improvement, paraesthesia, function, median nerve distal motor latencies, and sensory nerve conduction velocity) (appendices 8 and 9).^38^ ^39^ ^40^

### Lumbar spine decompression for spinal canal stenosis

Of 137 retrieved citations for lumbar spine decompression, 12 meta-analyses of randomised controlled trials were eligible for the review (fig 2; appendix 5). Publication dates ranged from 2011 to 2020, and AMSTAR scores ranged from 6 to 11 (appendices 6 and 7). Three meta-analyses compared surgical procedures (decompression, spinal fusion, interspinous process device implantation, and laminectomy) versus non-surgical treatment (orthosis, rehabilitation, physical therapy, exercise, heat and cold therapies, transcutaneous electrical nerve stimulation, ultrasonography, analgesics, non-steroidal anti-inflammatory drugs, epidural steroids, and cognitive-behavioural treatments), and nine meta-analyses compared two or more surgical or decompression procedures (decompression, spinal fusion, decompression plus fusion, interspinous process device implantation, discectomy, laminectomy, and laminotomy). The three meta-analyses comparing surgical treatment (mostly decompression procedures) with non-surgical treatment showed similar effects for operative and non-operative interventions (fig 3). The GRADE quality of the evidence was low.

For the comparisons among different surgical procedures, the results were generally consistent: no differences were seen in outcomes such as pain intensity, physical function or disability status, quality of life, recovery, perioperative blood loss, operation time, length of stay in hospital, and reoperation rates when the techniques were compared with each other. However, interspinous process device implantation seemed to be associated with higher reoperation rates than spinal decompression. No randomised controlled trials have compared lumbar spinal decompression with no treatment or placebo. In the single selected randomised controlled trial that represented the best body of evidence and compared segmental decompression with conservative treatment (non-steroidal anti-inflammatory drugs and physiotherapy), both treatment groups showed improvement during follow-up with no difference in walking ability (appendices 8 and 9).^41^

### Lumbar spine fusion for degenerative disc disease

Of 139 retrieved citations on lumbar spine fusion, 13 meta-analyses of randomised controlled trials were eligible for the review (fig 2; appendix 5). Publication dates ranged from 2010 to 2020, and AMSTAR scores ranged from 6 to 11 (appendices 6 and 7). Nine meta-analyses compared lumbar spine fusion with total disc replacement, two compared lumbar spine fusion with non-operative management (physical therapy, patient education, exercise, pain relief by acupuncture and injections), and two compared minimally invasive with open transforaminal lumbar interbody fusion for single level degenerative disease. The two meta-analyses comparing lumbar spine fusion with non-operative management both reported no differences in Oswestry Disability Index scores; however, one review reported that lumbar spine fusion was associated with surgical complications (fig 3).

For the comparison between lumbar spine fusion and total disc replacement, the results were variable in the earlier reviews. With the publication of newer randomised controlled trials, recent reviews showed that total disc replacement significantly improved pain and patient satisfaction, reduced reoperation rate and operation time, shortened duration of hospital admission, and decreased post-surgical complications compared with lumbar spine fusion in both the short term and the long term. The findings of the two reviews comparing minimally invasive versus open transforaminal lumbar interbody fusion for single level degenerative disease were discordant except for less blood loss and longer fluoroscopy time with the minimally invasive approach. On the basis of similarities in the research question and selection criteria, and differences in included trials, the Jadad decision algorithm indicated that the best available evidence should be chosen according to search strategies and application of selection criteria (fig 1). The best available evidence showed that the minimally invasive procedure was associated with less blood loss, shorter hospital stay, and slightly less disability.^42^ No randomised controlled trials have compared lumbar spine fusion with no treatment, placebo, or sham surgery. We selected two randomised controlled trials to represent the best body of evidence for lumbar spine fusion. Both trials compared lumbar spine fusion with cognitive intervention and exercises and showed no differences in success rates and return to work (appendices 8 and 9).^43^ ^44^

### Total hip replacement for end stage osteoarthritis

Of 168 retrieved articles, no reviews of randomised controlled trials comparing total hip replacement with no treatment, placebo, or sham surgery for the treatment of end stage osteoarthritis were identified (fig 2; appendix 5). We found no individual randomised controlled trials comparing total hip replacement versus non-surgical treatment. Three systematic reviews evaluated the clinical effectiveness of total hip replacement versus resurfacing arthroplasty for the treatment of end stage osteoarthritis of the hip (appendix 6). Their AMSTAR scores ranged from 4 to 6 (appendix 7). The evidence on functional outcomes, failure rate, and mortality was inconclusive; the risks of revision and component loosening were higher but the risk of implant dislocation was lower in patients receiving resurfacing arthroplasty compared with total hip replacement. The GRADE quality of the evidence ranged from very low to low.^45^ We also identified three individual randomised controlled trials that compared the effectiveness of resurfacing arthroplasty with that of total hip replacement.^46^ ^47^ ^48^ Except for a higher risk of infections among patients who had total hip replacement, evidence on all other outcomes was inconclusive.

### Total knee replacement for end stage osteoarthritis

Of 344 retrieved articles, no reviews of randomised controlled trials comparing total knee replacement versus no treatment, placebo, or sham surgery for the treatment of end stage osteoarthritis were identified (fig 2; appendix 5). We found one randomised controlled trial published in 2015, which compared total knee replacement followed by non-surgical treatment versus non-surgical treatment alone (exercise, education, dietary advice, use of insoles, and analgesics) in patients with moderate to severe knee osteoarthritis who were eligible for unilateral total knee replacement.^49^ Findings showed that treatment with total knee replacement followed by non-surgical treatment resulted in greater pain relief and functional improvement after 12 months compared with non-surgical treatment alone; however, total knee replacement was associated with a high number of serious adverse events (appendix 8; fig 3).

## Discussion

Using an umbrella review of systematic reviews and meta-analyses of randomised controlled trials, we sought to examine the body of evidence on the clinical effectiveness of 10 of the most elective common orthopaedic procedures and assess their impact on guideline recommendations. Of the 10 procedures, no randomised controlled trials have compared total hip replacement and meniscal repair for acute tears with non-operative care. The other eight procedures have been studied in such trials, and some evidence supports the superiority of total knee replacement and strongly supports carpal tunnel decompression over non-operative care. Randomised controlled trials have shown that arthroscopic anterior cruciate ligament reconstruction, arthroscopic partial meniscectomy, arthroscopic repair for acute rotator cuff tears, arthroscopic subacromial decompression, lumbar spinal decompression for spinal canal stenosis, and spinal fusion for degenerative disc disease have similar outcomes to non-operative care.

Comparison of our findings with the current recommendations and guidelines of national bodies is interesting (appendix 11). The American Academy of Orthopaedic Surgeons (AAOS) recommends using arthroscopic anterior cruciate ligament reconstruction in certain subgroups of patients,^50^ as well as using either rotator cuff repair or non-operative care for rotator cuff tears.^51^ The 2018 arthroscopic meniscal surgery treatment guidance from the British Association for Surgery of the Knee (BASK) recommends using arthroscopic meniscal repair to treat meniscal lesions of the knee in some patients,^52^ while acknowledging that no high quality level 1 evidence is available to support this. The NICE guideline recommendation is to use lumbar spine decompression when non-operative treatment has failed.^53^ NICE and other guideline bodies, on the basis of observational data, recommend total hip replacement for end stage osteoarthritis of the hip.^54^ ^55^ ^56^

Encouragingly, many of the guidelines from prominent national bodies closely reflect the current body of evidence. Consensus statements from guideline societies do not recommend arthroscopic partial meniscectomy in patients with knee pain and a meniscal tear,^57^ ^58^ strongly recommend against the use of subacromial decompression surgery for subacromial impingement syndrome (appendix 10),^35^ ^59^ ^60^ ^61^ support using open or endoscopic decompression for carpal tunnel syndrome,^62^ ^63^ recommend against lumbar spine decompression for people with low back pain,^53^ and recommend total knee replacement for end stage osteoarthritis of the knee. The evidence base for recommendations on total knee replacement was built wholly on observational retrospective studies that have often used prosthesis survival as the primary outcome measure.^64^ ^65^ However, this guidance is now supported by the subsequent randomised controlled trial published in 2015.^49^

Although seven of the procedures (namely, arthroscopic anterior cruciate ligament reconstruction for anterior cruciate ligament rupture, arthroscopic meniscal repair of the knee for traumatic tears, arthroscopic rotator cuff repair for acute rotator cuff tears, carpal tunnel decompression for carpal tunnel syndrome, lumbar spine decompression for spinal canal stenosis, and total hip replacement and total knee replacement for end stage osteoarthritis) have been recommended for use by national guidelines, a high quality body of evidence on the clinical effectiveness to definitively support these recommendations does not exist for most of these procedures. This is mainly a consequence of the lack of randomised controlled trials that compare the procedure with non-operative care. The evidence base for the recommendations was mostly built on comparisons involving two or more different techniques of the same procedure (for example, endoscopic versus open), observational retrospective studies and case series with no control groups, and expert opinions. For example, the recommendations for arthroscopic meniscal repair were based on mostly indirect evidence and low quality observational studies,^52^ and the guideline group highlighted this as a priority area for further research.^52^ For total hip replacement, the evidence for the recommendations was based on head-to-head comparisons between different types of hip prosthesis and uncontrolled studies that have used prosthesis survival as the primary outcome measure.^56^ This lack of randomised controlled trials evidence does not mean that the interventions are ineffective, but without evidence from randomised controlled trials, disentangling regression to the mean, surgical placebo, and the true treatment effect is extremely difficult.

### Strengths and limitations of study

The strengths of this study include its novelty and the comprehensive search of well known databases as well as guidelines. Although umbrella reviews provide top level evidence and important insights on the clinical effectiveness of interventions, several inherent limitations to our review deserve consideration. Because of the absence of meta-analyses on a few procedures, due to the lack of randomised controlled trials, we used relevant study designs according to the hierarchy of evidence. We used the UK NICE guidelines evidence search for recommendations on each procedure, given that the study setting was the UK. The recommendations can be applied to other healthcare settings, however, as our search yielded guidelines from elsewhere in Europe and the US. Several meta-analyses did not use GRADE scores to appraise the quality of findings. Finally, several of the meta-analyses included in our review scored low when appraised with the AMSTAR tool, showing the need for future reviews to use accepted methods of reporting.

### Explanations for findings

Surgery is expensive and associated with considerable morbidity, increased risk of complications attributed to the surgical intervention, and excess mortality. Seeking high level evidence to support surgery is thus imperative. When high level evidence shows that non-operative care is equivalent, surgeons and patients should carefully consider what would be achieved by doing surgery. A cogent argument can be made for surgery being used as a second line treatment when non-surgical measures have failed or in certain subgroups of patients who have been identified as “responders” to surgical treatment. However, trials first need to be done to show the efficacy of surgery in these scenarios and to define the period of treatment with non-surgical interventions that is appropriate before surgery is undertaken. For an operation such as total hip replacement, the understandable assumption is made that all improvement is due to the surgery, but some may be due to the natural history including regression to the mean or treatment effect from non-replacement co-interventions. Given the current waiting lists for surgery due to the covid-19 pandemic, a randomised controlled trial comparing total hip replacement with best non-surgical care may be timely, especially if those patients randomised to non-operative care are subsequently offered surgery should they be dissatisfied with the outcome of non-operative care.

Some of these interventions are just not clinically effective or may be effective only when used in specific circumstances. For example, although the use of arthroscopic partial meniscectomy in patients with knee pain and a meniscal tear is not recommended, especially in patients with significant or end stage osteoarthritis, guidelines suggest that the procedure can be used for a specific type of meniscal tear and should be used only in patients who have not responded to a period of non-surgical treatment. Despite the good body of evidence on the clinical ineffectiveness of arthroscopic subacromial decompression, national clinical guidelines recommend its use for patients with pure subacromial shoulder impingement whose symptoms fail to resolve with adequate non-operative treatment.^61^

The observation that most commonly used and recommended orthopaedic procedures had a limited and low quality evidence base relating to their effectiveness is concerning. Why clinicians and healthcare systems would choose to offer these procedures with limited evidence of their clinical effectiveness may not be entirely clear but is partly justifiable. Important reasons include the lack of randomised controlled trials that compare the procedure with no treatment or placebo. Properly conducted definitive trials are more difficult to run with orthopaedic interventions than with drugs and other interventions.^8^ They are labour intensive and expensive, and they have a late response given the demand for long term follow-up and the potential for crossover between arms; hence, most studies are based on small case series. Problems with recruitment, blinding, and quality of reporting also contribute to the low quality standards of some of the orthopaedic trials contributing to this review. However, the large number of recent high quality orthopaedic randomised controlled trials being conducted in the UK is extremely encouraging,^66^ ^67^ ^68^ and the orthopaedic community should thus be lauded for its efforts. Another important reason for the lack of evidence is the failure of investigators to report their negative findings, the refusal of journals to publish such findings, or both. Although no-treatment or placebo controlled trials are feasible for some orthopaedic interventions, they may not be feasible for all. Some of the guideline bodies highlighted the need to prioritise research so that some of these procedures, such as arthroscopic meniscal repair of the knee for traumatic tears, would be informed by a good evidence base. Furthermore, interventions may work, even if the evidence base has not yet been established, or the observational evidence may be so overwhelming that randomised controlled trials would be deemed unethical or redundant. Hip replacement may be an example of this.

### Implications of findings

With these findings, a need exists for an improved and more rigorous approach to the recommendation of procedures with limited evidence on their clinical effectiveness. Over the past few years, NHS England, in partnership with NHS Clinical Commissioners, the Academy of Royal Medical Colleges, and NICE, has put forward proposals to stop or reduce the commissioning of various interventions that have inconclusive evidence for their clinical effectiveness; the goal of this is to improve safety for patients and curb unnecessary use of time and resources.^61^ ^69^ ^70^ On the basis of clinical evidence from various guidelines, including from NICE, 17 interventions have been selected and have been categorised as those that should not be routinely commissioned or performed and those that should be routinely commissioned or performed only when specific criteria are met.^70^ Featuring in the list were several orthopaedic procedures including arthroscopic subacromial decompression and carpal tunnel decompression. Of the 10 procedures that we studied, carpal tunnel decompression had the strongest evidence base supporting it. An urgent need exists to prioritise research, especially for the procedures with a limited evidence base, and for definitive randomised controlled trial designs to evaluate their clinical effectiveness. This will improve patient care, cut healthcare costs, permit more efficient use of our resources, and increase societal trust in orthopaedic interventions. A recent comment in *The BMJ* suggested that we should use “impactful, evidence based surgery alongside impactful, evidence based non-surgical care that may be faster, safer, and delivered at a lower cost.” Furthermore, with 10 million patients in England awaiting surgery, “now is an ideal time to invest in large platform trials.”^7^ The findings of this study support this view.

### Conclusions

This umbrella review of the body of evidence on the 10 most common elective orthopaedic procedures suggests that most of these procedures recommended by national guidelines and used by surgeons have insufficient readily available, high quality evidence on their clinical effectiveness, which is mainly because of a lack of definitive trials. This forces patients and clinicians to make decisions based solely on observational evidence. When randomised controlled trials have been conducted, they sometimes support observational evidence and expert opinion, but not universally so. To optimise decision making, surgeons should apprise themselves of the evidence, make decisions based on the highest quality trials available, and, in the absence of these, base their judgment on observational evidence, acknowledging that this may be imperfect. Simultaneously, surgeons and research funding bodies should actively contribute to filling the key knowledge gaps by enabling and participating in well constructed pragmatic randomised controlled trials.

## What is already known on this topic

The burden of musculoskeletal conditions on health services is large, with more than one million patients undergoing surgical interventions according to Hospital Episode Statistics
The National Institute for Health and Care Excellence, NHS, and British Orthopaedic Association produce national clinical guidelines recommending common elective orthopaedic interventions
These recommendations often lack strong supporting evidence, particularly in the form of randomised controlled trials

## What this study adds

Most common elective orthopaedic interventions are not backed up by readily available high quality evidence, mostly owing to a lack of definitive randomised controlled trials
Of the procedures studied, carpal tunnel decompression and total knee replacement showed superiority over non-operative care
An urgent need exists to prioritise research into common elective orthopaedic interventions compared with no treatment, placebo, and non-operative treatment

## Data Availability

Data sharing: Data extracted from individual papers are available from the corresponding author.

## Web extra

Extra material supplied by authors

Web appendix: Supplementary materials

## Notes

The lead author affirms that this manuscript is an honest, accurate, and transparent account of the study being reported; that no important aspects of the study have been omitted; and that any discrepancies from the study as planned (and, if relevant, registered) have been explained.

Dissemination to participants and related patient and public communities: We plan to disseminate the findings to patient groups, healthcare professionals, academics, and relevant stakeholders and professional bodies such as NICE, NHS Clinical Commissioners, and the British Orthopaedic Association. Findings will reach patient networks with the help of our patient and public involvement group. Other dissemination portals will include presentations at key conferences, workshops, websites, and policy briefings.

Provenance and peer review: Not commissioned; externally peer reviewed.
