# Supplementary material for: Common elective orthopaedic procedures and their clinical effectiveness: umbrella review of level 1 evidence
Source: BMJ. 2021 Jul 8;374:n1511. doi: 10.1136/bmj.n1511 (PMC8262448; doi:10.1136/bmj.n1511)
Supplement: Supplementary file 1 — Web appendix: Supplementary materials [file bloa063464.ww1.pdf]

Supplementary Material

Index

**Appendix 1**	PRISMA checklist
**Appendix 2**	MOOSE checklist
**Appendix 3**	Search strategy for each procedure
**Appendix 4**	Hierarchy of evidence
**Appendix 5**	Reference list of included studies
**Appendix 6**	Characteristics of included meta-analyses or relevant studies
**Appendix 7**	AMSTAR scores for the included studies
**Appendix 8**	Findings of randomised controlled trials at low risk of bias
**Appendix 9**	Reference list of randomised controlled trials at low risk of bias
**Appendix 10**	Major guideline recommendations on subacromial decompression surgery
**Appendix 11**	Common orthopaedic procedures, body of evidence and recommendations by clinical guidelines

Appendix 1. PRISMA checklist

Section/topic	Item No	Checklist item	Reported on page number/section name
**Title**			
Title	1	Identify the report as a systematic review, meta-analysis, or both	1
**Abstract**			
Structured summary	2	Provide a structured summary including, as applicable, background, objectives, data sources, study eligibility criteria, participants, interventions, study appraisal and synthesis methods, results, limitations, conclusions and implications of key findings, systematic review registration number	2
**Introduction**			
Rationale	3	Describe the rationale for the review in the context of what is already known	Introduction
Objectives	4	Provide an explicit statement of questions being addressed with reference to participants, interventions, comparisons, outcomes, and study design (PICOS)	Introduction
**Methods**			
Protocol and registration	5	Indicate if a review protocol exists, if and where it can be accessed (such as web address), and, if available, provide registration information including registration number	Methods
Eligibility criteria	6	Specify study characteristics (such as PICOS, length of follow-up) and report characteristics (such as years considered, language, publication status) used as criteria for eligibility, giving rationale	Methods
Information sources	7	Describe all information sources (such as databases with dates of coverage, contact with study authors to identify additional studies) in the search and date last searched	Methods
Search	8	Present full electronic search strategy for at least one database, including any limits used, such that it could be repeated	Appendix 3
Study selection	9	State the process for selecting studies (that is, screening, eligibility, included in systematic review, and, if applicable, included in the meta-analysis)	Methods
Data collection process	10	Describe method of data extraction from reports (such as piloted forms, independently, in duplicate) and any processes for obtaining and confirming data from investigators	Methods
Data items	11	List and define all variables for which data were sought (such as PICOS, funding sources) and any assumptions and simplifications made	Methods
Risk of bias in individual studies	12	Describe methods used for assessing risk of bias of individual studies (including specification of whether this was done at the study or outcome level), and how this information is to be used in any data synthesis	Methods
Summary measures	13	State the principal summary measures (such as risk ratio, difference in means).	Methods
Synthesis of results	14	Describe the methods of handling data and combining results of studies, if done, including measures of consistency (such as I^2 statistic) for each meta-analysis	Methods
Risk of bias across studies	15	Specify any assessment of risk of bias that may affect the cumulative evidence (such as publication bias, selective reporting within studies)	Methods
Additional analyses	16	Describe methods of additional analyses (such as sensitivity or subgroup analyses, meta-regression), if done, indicating which were pre-specified	Methods
**Results**			
Study selection	17	Give numbers of studies screened, assessed for eligibility, and included in the review, with reasons for exclusions at each stage, ideally with a flow diagram	Results; Appendix 6
Study characteristics	18	For each study, present characteristics for which data were extracted (such as study size, PICOS, follow-up period) and provide the citations	Results; Table 2
Risk of bias within studies	19	Present data on risk of bias of each study and, if available, any outcome-level assessment (see item 12).	Results; Table 3
Results of individual studies	20	For all outcomes considered (benefits or harms), present for each study (a) simple summary data for each intervention group and (b) effect estimates and confidence intervals, ideally with a forest plot	Results; Table 2
Synthesis of results	21	Present results of each meta-analysis done, including confidence intervals and measures of consistency	Results; Table 2
Risk of bias across studies	22	Present results of any assessment of risk of bias across studies (see item 15)	Results; Table 3
Additional analysis	23	Give results of additional analyses, if done (such as sensitivity or subgroup analyses, meta-regression) (see item 16)	Not applicable
**Discussion**			
Summary of evidence	24	Summarise the main findings including the strength of evidence for each main outcome; consider their relevance to key groups (such as health care providers, users, and policy makers)	Discussion
Limitations	25	Discuss limitations at study and outcome level (such as risk of bias), and at review level (such as incomplete retrieval of identified research, reporting bias)	Discussion
Conclusions	26	Provide a general interpretation of the results in the context of other evidence, and implications for future research	Discussion
**Funding**			
Funding	27	Describe sources of funding for the systematic review and other support (such as supply of data) and role of funders for the systematic review	Discussion

Appendix 2. MOOSE checklist

Criteria		Brief description of how the criteria were handled in the review
**Reporting of background**		
√	Problem definition	Recommendations in clinical guidelines for common orthopaedic interventions often lack strong supporting evidence, particularly in the form of clinical trials. It is concerning that orthopaedic interventions and prostheses do not have readily available evidence to support their use. In this context, we have carried out an umbrella review (systematic review and meta-analysis) to evaluate the body of evidence behind ten of the commonest orthopaedic surgical interventions.
√	Hypothesis statement	There is not a strong evidence base to support many commonly performed orthopaedic procedures.
√	Description of study outcomes	The quality and quantity of the evidence behind the commonest orthopaedic interventions, and the strength of the recommendations in relevant national clinical guidelines
√	Type of exposure	Arthroscopic anterior cruciate ligament reconstruction, arthroscopic meniscal repair of the knee, arthroscopic partial meniscectomy of the knee, arthroscopic rotator cuff repair, arthroscopic subacromial decompression, carpal tunnel decompression, lumbar spine decompression, lumbar spine fusion, total hip replacement, and total knee replacement
√	Type of study designs used	Comparative observational studies and randomised controlled trials
√	Study population	Patients undergoing the aforementioned exposures, compared to patients undergoing no treatment/placebo/nonoperative treatment
**Reporting of search strategy should include**		
√	Qualifications of searchers	Setor K Kunutsor, PhD; Richard L Donovan, MSc
√	Search strategy, including time period included in the synthesis and keywords	Time period: from inception to September 2020<br>The detailed search strategy can be found in Appendix 3
√	Databases and registries searched	MEDLINE, EMBASE, Web of Science, and Cochrane databases
√	Search software used, name and version, including special features	OvidSP was used to search EMBASE and MEDLINE<br>EndNote X9 used to manage references
√	Use of hand searching	We searched bibliographies of retrieved papers
√	List of citations located and those excluded, including justifications	Details of the literature search process are outlined in Appendix 6. The citation list for excluded studies is available on request.
√	Method of addressing articles published in languages other than English	Not applicable
√	Method of handling abstracts and unpublished studies	Abstracts with no full-text publications were not included.
√	Description of any contact with authors	None
**Reporting of methods should include**		
√	Description of relevance or appropriateness of studies assembled for assessing the hypothesis to be tested	Detailed inclusion and exclusion criteria are described in the Methods section.
√	Rationale for the selection and coding of data	Data extracted from each of the studies were relevant to the population characteristics, study design, exposure, and outcome.
√	Assessment of confounding	We assessed confounding by ranking individual studies based on different adjustment levels and performed subgroup analyses to evaluate differences in the overall estimates according to levels of adjustment.
√	Assessment of study quality, including blinding of quality assessors; stratification or regression on possible predictors of study results	Study quality was assessed based on the eleven-item Assessment of Multiple Systematic Reviews (AMSTAR) instrument, which includes ratings for quality in the search, analysis, and transparency of a meta-analysis. It has good reliability and external validity.
√	Assessment of heterogeneity	The heterogeneity of the studies was quantified with I^2 statistic that provides the relative amount of variance of the summary effect due to the between-study heterogeneity.
√	Description of statistical methods in sufficient detail to be replicated	Details are described in the Methods section.
√	Provision of appropriate tables and graphics	Tables 1-3; Appendices 3-10
**Reporting of results should include**		
√	Graph summarizing individual study estimates and overall estimate	N/a
√	Table giving descriptive information for each study included	Table 2
√	Results of sensitivity testing	Table 3
√	Indication of statistical uncertainty of findings	N/a

Reporting of discussion should include		
√	Quantitative assessment of bias	Study quality was assessed based on the eleven-item Assessment of Multiple Systematic Reviews (AMSTAR) instrument, which includes ratings for quality in the search, analysis, and transparency of a meta-analysis. It has good reliability and external validity.
√	Justification for exclusion	All studies were excluded based on the pre-defined inclusion criteria in the Methods section.
√	Assessment of quality of included studies	This is discussed included in the Methods section.
**Reporting of conclusions should include**		
√	Consideration of alternative explanations for observed results	This is described in the Discussion section.
√	Generalization of the conclusions	This is discussed in the context of the results.
√	Guidelines for future research	We recommend definitive randomised controlled trials are required to vastly improve the quality of orthopaedic research.
√	Disclosure of funding source	Provided.

Appendix 3. Search strategy for each procedure

Arthroscopic anterior cruciate ligament reconstruction

1 exp Arthroscopy/ or arthroscopic.mp. (31580)
2 exp Anterior Cruciate Ligament Reconstruction/ (5032)
3 ACL reconstruction.mp. (7040)
4 ACLR.mp. (1197)
5 (((comprehensive* or integrative or systematic*) adj3 (bibliographic* or review* or literature)) or (meta-analy* or metaanaly* or "research synthesis" or ((information or data) adj3 synthesis) or (data adj2 extract*))).ti,ab. or (cinahl or (cochrane adj3 trial*) or embase or medline or psyclit or (psycinfo not "psycinfo database") or pubmed or scopus or "sociological abstracts" or "web of science").ab. or ("cochrane database of systematic reviews" or evidence report technology assessment or evidence report technology assessment summary).jn. or Evidence Report: Technology Assessment*.jn. or ((review adj5 (rationale or evidence)).ti,ab. and review.pt.) or meta-analysis as topic/ or Meta-Analysis.pt. (457870)
6 2 or 3 or 4 (9115)
7 1 and 5 and 6 (102)
8 limit 7 to (english language and humans) (90)

Arthroscopic meniscal repair of the knee

1 exp Arthroscopy/ or arthroscopic.mp. (31580)
2 meniscus repair.mp. (461)
3 meniscal repair.mp. (991)
4 meniscal surgery.mp. (209)
5 exp Knee/ or exp Knee Joint/ (71155)
6 (((comprehensive* or integrative or systematic*) adj3 (bibliographic* or review* or literature)) or (meta-analy* or metaanaly* or "research synthesis" or ((information or data) adj3 synthesis) or (data adj2 extract*))).ti,ab. or (cinahl or (cochrane adj3 trial*) or embase or medline or psyclit or (psycinfo not "psycinfo database") or pubmed or scopus or "sociological abstracts" or "web of science").ab. or ("cochrane database of systematic reviews" or evidence report technology assessment or evidence report technology assessment summary).jn. or Evidence Report: Technology Assessment*.jn. or ((review adj5 (rationale or evidence)).ti,ab. and review.pt.) or meta-analysis as topic/ or Meta-Analysis.pt. (457870)
7 2 or 3 or 4 (1467)
8 1 and 5 and 6 and 7 (25)
9 limit 8 to (english language and humans) (25)

Arthroscopic partial meniscectomy of the knee

1 exp Arthroscopy/ or arthroscopic.mp. (31580)
2 exp Meniscectomy/ (240)
3 menisc*.mp. (18214)
4 exp Knee/ or exp Knee Joint/ (71155)
5 (((comprehensive* or integrative or systematic*) adj3 (bibliographic* or review* or literature)) or (meta-analy* or metaanaly* or "research synthesis" or ((information or data) adj3 synthesis) or (data adj2 extract*))).ti,ab. or (cinahl or (cochrane adj3 trial*) or embase or medline or psyclit or (psycinfo not "psycinfo database") or pubmed or scopus or "sociological abstracts" or "web of science").ab. or ("cochrane database of systematic reviews" or evidence report technology assessment or evidence report technology assessment summary).jn. or Evidence Report: Technology Assessment*.jn. or ((review adj5 (rationale or evidence)).ti,ab. and review.pt.) or meta-analysis as topic/ or Meta-Analysis.pt. (457870)
6 2 or 3 (18214)
7 1 and 4 and 5 and 6 (79)
8 limit 7 to (english language and humans) (78)

Arthroscopic rotator cuff repair

1 exp Arthroscopy/ or arthroscop*.mp. (36005)
2 exp Rotator Cuff/ (6447)
3 (((comprehensive* or integrative or systematic*) adj3 (bibliographic* or review* or literature)) or (meta-analy* or metaanaly* or "research synthesis" or ((information or data) adj3 synthesis) or (data adj2 extract*))).ti,ab. or (cinahl or (cochrane adj3 trial*) or embase or medline or psyclit or (psycinfo not "psycinfo database") or pubmed or scopus or "sociological abstracts" or "web of science").ab. or ("cochrane database of systematic reviews" or evidence report technology assessment or evidence report technology assessment summary).jn. or Evidence Report: Technology Assessment*.jn. or ((review adj5 (rationale or evidence)).ti,ab. and review.pt.) or meta-analysis as topic/ or Meta-Analysis.pt. (456965)
4 1 and 2 and 3 (115)
5 limit 4 to (english language and humans) (110)

Arthroscopic subacromial decompression

1 Arthroscopy/ or arthroscop*.mp. (35699)
2 exp Decompression/ (2747)
3 subacromial.mp. (2758)
4 exp Shoulder/ (12902)
5 exp Shoulder Impingement Syndrome/ (1765)
6 subacromial impingement syndrome.mp. (408)
7 (((comprehensive* or integrative or systematic*) adj3 (bibliographic* or review* or literature)) or (meta-analy* or metaanaly* or "research synthesis" or ((information or data) adj3 synthesis) or (data adj2 extract*))).ti,ab. or (cinahl or (cochrane adj3 trial*) or embase or medline or psyclit or (psycinfo not "psycinfo database") or pubmed or scopus or "sociological abstracts" or "web of science").ab. or ("cochrane database of systematic reviews" or evidence report technology assessment or evidence report technology assessment summary).jn. or Evidence Report: Technology Assessment*.jn. or ((review adj5 (rationale or evidence)).ti,ab. and review.pt.) or meta-analysis as topic/ or Meta-Analysis.pt. (451769)
8 1 or 2 or 3 or 4 (52178)
9 5 or 6 (1882)
10 7 and 8 and 9 (79)
11 limit 10 to (english language and humans) (71)

Carpal tunnel decompression

1 carpal tunnel surgery.mp. (283)
2 carpal tunnel release.mp. (1675)
3 exp Decompression, Surgical/ (30551)
4 exp Carpal Tunnel Syndrome/ (8591)
5 (((comprehensive* or integrative or systematic*) adj3 (bibliographic* or review* or literature)) or (meta-analy* or metaanaly* or "research synthesis" or ((information or data) adj3 synthesis) or (data adj2 extract*))).ti,ab. or (cinahl or (cochrane adj3 trial*) or embase or medline or psyclit or (psycinfo not "psycinfo database") or pubmed or scopus or "sociological abstracts" or "web of science").ab. or ("cochrane database of systematic reviews" or evidence report technology assessment or evidence report technology assessment summary).jn. or Evidence Report: Technology Assessment*.jn. or ((review adj5 (rationale or evidence)).ti,ab. and review.pt.) or meta-analysis as topic/ or Meta-Analysis.pt. (451321)
6 1 or 2 or 3 (32131)
7 4 and 5 and 6 (53)
8 limit 7 to (english language and humans) (49)

Lumbar spine decompression

1 exp Decompression, Surgical/ or lumbar decompression.mp. (30909)
2 spinal decompression.mp. (640)
3 lumbar spinal decompression.mp. (42)
4 stenosis.mp. (194098)
5 (((comprehensive* or integrative or systematic*) adj3 (bibliographic* or review* or literature)) or (meta-analy* or metaanaly* or "research synthesis" or ((information or data) adj3 synthesis) or (data adj2 extract*))).ti,ab. or (cinahl or (cochrane adj3 trial*) or embase or medline or psyclit or (psycinfo not "psycinfo database") or pubmed or scopus or "sociological abstracts" or "web of science").ab. or ("cochrane database of systematic reviews" or evidence report technology assessment or evidence report technology assessment summary).jn. or Evidence Report: Technology Assessment*.jn. or ((review adj5 (rationale or evidence)).ti,ab. and review.pt.) or meta-analysis as topic/ or Meta-Analysis.pt. (456914)
6 1 or 2 or 3 (31243)
7 4 and 5 and 6 (128)

Lumbar spine fusion

1 exp Spinal Fusion/ (25464)
2 exp Lumbar Vertebrae/ or lumbar.mp. (122546)
3 exp Intervertebral Disc Degeneration/ or degenerative dis*.mp. (20491)
4 (((comprehensive* or integrative or systematic*) adj3 (bibliographic* or review* or literature)) or (meta-analy* or metaanaly* or "research synthesis" or ((information or data) adj3 synthesis) or (data adj2 extract*))).ti,ab. or (cinahl or (cochrane adj3 trial*) or embase or medline or psyclit or (psycinfo not "psycinfo database") or pubmed or scopus or "sociological abstracts" or "web of science").ab. or ("cochrane database of systematic reviews" or evidence report technology assessment or evidence report technology assessment summary).jn. or Evidence Report: Technology Assessment*.jn. or ((review adj5 (rationale or evidence)).ti,ab. and review.pt.) or meta-analysis as topic/ or Meta-Analysis.pt. (457475)
5 1 and 2 and 3 and 4 (124)
6 limit 5 to (english language and humans) (118)

**Total hip replacement**
1 exp Arthroplasty, Replacement, Hip/ (27189)<br>2 exp Osteoarthritis/ (63224)<br>3 (((comprehensive* or integrative or systematic*) adj3 (bibliographic* or review* or literature)) or (meta-analy* or metaanaly* or "research synthesis" or ((information or data) adj3 synthesis) or (data adj2 extract*))).ti,ab. or (cinahl or (cochrane adj3 trial*) or embase or medline or psyclit or (psycinfo not "psycinfo database") or pubmed or scopus or "sociological abstracts" or "web of science").ab. or ("cochrane database of systematic reviews" or evidence report technology assessment or evidence report technology assessment summary).jn. or Evidence Report: Technology Assessment*.jn. or ((review adj5 (rationale or evidence)).ti,ab. and review.pt.) or meta-analysis as topic/ or Meta-Analysis.pt. (457475)<br>4 1 and 2 and 3 (145)<br>5 limit 4 to (english language and humans) (135)

**Total knee replacement**
1 exp Arthroplasty, Replacement, Knee/ (23882)<br>2 exp Osteoarthritis/ (63224)<br>3 (((comprehensive* or integrative or systematic*) adj3 (bibliographic* or review* or literature)) or (meta-analy* or metaanaly* or "research synthesis" or ((information or data) adj3 synthesis) or (data adj2 extract*))).ti,ab. or (cinahl or (cochrane adj3 trial*) or embase or medline or psyclit or (psycinfo not "psycinfo database") or pubmed or scopus or "sociological abstracts" or "web of science").ab. or ("cochrane database of systematic reviews" or evidence report technology assessment or evidence report technology assessment summary).jn. or Evidence Report: Technology Assessment*.jn. or ((review adj5 (rationale or evidence)).ti,ab. and review.pt.) or meta-analysis as topic/ or Meta-Analysis.pt. (457475)<br>4 1 and 2 and 3 (320)<br>5 limit 4 to (english language and humans) (303)

Appendix 4. Hierarchy of evidence

**1a**	Systematic reviews of randomised controlled trials
**1b**	Individual randomised controlled trials
**1c**	All or none randomised controlled trials
**2a**	Systematic reviews of cohort studies
**2b**	Individual cohort study or low quality randomised controlled trials
**2c**	Outcomes' research; ecological studies
**3a**	Systematic review of case-control studies
**3b**	Individual case-control study
**4**	Case series
**5**	Expert opinion without explicit critical appraisal/pre-clinical biomechanical data

Appendix 5. Reference list of included studies

1. Abram SGF, Hopewell S, Monk AP, Bayliss LE, Beard DJ, Price AJ. Arthroscopic partial meniscectomy for meniscal tears of the knee: a systematic review and meta-analysis. *Br J Sports Med*. 2020;54(11):652-63.
2. Bai DY, Liang L, Zhang BB, *et al*. Total disc replacement versus fusion for lumbar degenerative diseases - a meta-analysis of randomized controlled trials. *Medicine (Baltimore)*. 2019;98(29):e16460.
3. Brignardello-Petersen R, Guyatt GH, Buchbinder R, *et al*. Knee arthroscopy versus conservative management in patients with degenerative knee disease: a systematic review. *BMJ Open*. 2017;7(5):e016114.
4. Bydon M, De la Garza-Ramos R, Macki M, Baker A, Gokaslan AK, Bydon A. Lumbar fusion versus nonoperative management for treatment of discogenic low back pain: a systematic review and meta-analysis of randomized controlled trials. *J Spinal Disord Tech*. 2014;27(5):297-304.
5. Chen L, Duan X, Huang X, Lv J, Peng K, Xiang Z. Effectiveness and safety of endoscopic versus open carpal tunnel decompression. *Arch Orthop Trauma Surg*. 2014;134(4):585-93.
6. Hiratzka J, Rastegar F, Contag AG, Norvell DC, Anderson PA, Hart RA. Adverse Event Recording and Reporting in Clinical Trials Comparing Lumbar Disk Replacement with Lumbar Fusion: A Systematic Review. *Global Spine J*. 2015;5(6):486-95.
7. Hu K, Zhang T, Xu W. Intraindividual comparison between open and endoscopic release in bilateral carpal tunnel syndrome: a meta-analysis of randomized controlled trials. *Brain Behav*. 2016;6(3):e00439.
8. Jacobs W, Van der Gaag NA, Tuschel A, *et al*. Total disc replacement for chronic back pain in the presence of disc degeneration. *Cochrane Database Syst Rev*. 2012(9):CD008326.
9. Ji X, Bi C, Wang F, Wang Q. Arthroscopic versus mini-open rotator cuff repair: an up-to-date meta-analysis of randomized controlled trials. *Arthroscopy*. 2015;31(1):118-24.
10. Jiang Y, Zhang K, Die J, Shi Z, Zhao H, Wang K. A systematic review of modern metal-on-metal total hip resurfacing vs standard total hip arthroplasty in active young patients. *J Arthroplasty*. 2011;26(3):419-26.
11. Karjalainen TV, Jain NB, Heikkinen J, Johnston RV, Page CM, Buchbinder R. Surgery for rotator cuff tears. *Cochrane Database Syst Rev*. 2019;12:CD013502.
12. Karjalainen TV, Jain NB, Page CM, *et al*. Subacromial decompression surgery for rotator cuff disease. *Cochrane Database Syst Rev*. 2019;1:CD005619.
13. Khan M, Evaniew N, Bedi A, Ayeni OR, Bhandari M. Arthroscopic surgery for degenerative tears of the meniscus: a systematic review and meta-analysis. *CMAJ*. 2014;186(14):1057-64.
14. Kovacs FM, Urrutia G, Alarcon JD. Surgery versus conservative treatment for symptomatic lumbar spinal stenosis: a systematic review of randomized controlled trials. *Spine (Phila Pa 1976)*. 2011;36(20):E1335-51.
15. Lahdeoja T, Karjalainen T, Jokihaara J, *et al*. Subacromial decompression surgery for adults with shoulder pain: a systematic review with meta-analysis. *Br J Sports Med*. 2020;54(11):665-73.
16. Lee DY, Park YJ, Kim HJ, *et al*. Arthroscopic meniscal surgery versus conservative management in patients aged 40 years and older: a meta-analysis. *Arch Orthop Trauma Surg*. 2018;138(12):1731-9.
17. Li A, Li X, Zhong Y. Is minimally invasive superior than open transforaminal lumbar interbody fusion for single-level degenerative lumbar diseases: a meta-analysis. *J Orthop Surg Res*. 2018;13(1):241.
18. Li G, Kong L, Kou N, *et al*. The comparison of limited-incision versus standard-incision in treatment of carpal tunnel syndrome: A meta-analysis of randomized controlled trials. *Medicine (Baltimore)*. 2019;98(18):e15372.
19. Li M, Yang H, Wang G. Interspinous process devices for the treatment of neurogenic intermittent claudication: a systematic review of randomized controlled trials. *Neurosurg Rev*. 2017;40(4):529-36.
20. Li YZ, Sun P, Chen D, Tang L, Chen CH, Wu AM. Artificial Total Disc Replacement Versus Fusion for Lumbar Degenerative Disc Disease: An Update Systematic Review and Meta-Analysis. *Turk Neurosurg*. 2020;30(1):1-10.
21. Lien-Iversen T, Morgan DB, Jensen C, Risberg MA, Engebretsen L, Viberg B. Does surgery reduce knee osteoarthritis, meniscal injury and subsequent complications compared with non-surgery after ACL rupture with at least 10 years follow-up? A systematic review and meta-analysis. *Br J Sports Med*. 2020;54(10):592-8.
22. Ma XL, Zhao XW, Ma JX, Li F, Wang Y, Lu B. Effectiveness of surgery versus conservative treatment for lumbar spinal stenosis: A system review and meta-analysis of randomized controlled trials. *Int J Surg*. 2017;44:329-38.
23. Machado GC, Ferreira PH, Yoo RI, *et al*. Surgical options for lumbar spinal stenosis. *Cochrane Database Syst Rev*. 2016;11:CD012421.
24. Miller LE, Bhattacharyya S, Pracyk J. Minimally Invasive Versus Open Transforaminal Lumbar Interbody Fusion for Single-Level Degenerative Disease: A Systematic Review and Meta-Analysis of Randomized Controlled Trials. *World Neurosurg*. 2020;133:358-65 e4.
25. Mo Z, Zhang R, Chang M, Tang S. Exercise therapy versus surgery for lumbar spinal stenosis: A systematic review and meta-analysis. *Pak J Med Sci*. 2018;34(4):879-85.
26. Monk AP, Davies LJ, Hopewell S, Harris K, Beard DJ, Price AJ. Surgical versus conservative interventions for treating anterior cruciate ligament injuries. *Cochrane Database Syst Rev*. 2016;4:CD011166.
27. Nazari G, MacDermid JC, Bryant D, Athwal GS. The effectiveness of surgical vs conservative interventions on pain and function in patients with shoulder impingement syndrome. A systematic review and meta-analysis. *PLoS One*. 2019;14(5):e0216961.
28. Nie H, Chen G, Wang X, Zeng J. Comparison of Total Disc Replacement with lumbar fusion: a meta-analysis of randomized controlled trials. *J Coll Physicians Surg Pak*. 2015;25(1):60-7.

29. Overdevest GM, Jacobs W, Vleggeert-Lankamp C, Thome C, Gunzburg R, Peul W. Effectiveness of posterior decompression techniques compared with conventional laminectomy for lumbar stenosis. *Cochrane Database Syst Rev*. 2015(3):CD010036.
30. Poetscher AW, Gentil AF, Ferretti M, Lenza M. Interspinous process devices for treatment of degenerative lumbar spine stenosis: A systematic review and meta-analysis. *PLoS One*. 2018;13(7):e0199623.
31. Rao MJ, Cao SS. Artificial total disc replacement versus fusion for lumbar degenerative disc disease: a meta-analysis of randomized controlled trials. *Arch Orthop Trauma Surg*. 2014;134(2):149-58.
32. Sanati KA, Mansouri M, Macdonald D, Ghafghazi S, Macdonald E, Yadegarfar G. Surgical techniques and return to work following carpal tunnel release: a systematic review and meta-analysis. *J Occup Rehabil*. 2011;21(4):474-81.
33. Sayegh ET, Strauch RJ. Open versus endoscopic carpal tunnel release: a meta-analysis of randomized controlled trials. *Clin Orthop Relat Res*. 2015;473(3):1120-32.
34. Schemitsch C, Chahal J, Vicente M, *et al*. Surgical repair versus conservative treatment and subacromial decompression for the treatment of rotator cuff tears: a meta-analysis of randomized trials. *Bone Joint J*. 2019;101-B(9):1100-6.
35. Shen J, Xu S, Xu S, Ye S, Hao J. Fusion or Not for Degenerative Lumbar Spinal Stenosis: A Meta-Analysis and Systematic Review. *Pain Physician*. 2018;21(1):1-8.
36. Skou ST, Roos EM, Laursen MB, *et al*. A Randomized, Controlled Trial of Total Knee Replacement. *N Engl J Med*. 2015;373(17):1597-606.
37. Smith TO, Nichols R, Donell ST, Hing CB. The clinical and radiological outcomes of hip resurfacing versus total hip arthroplasty: a meta-analysis and systematic review. *Acta Orthop*. 2010;81(6):684-95.
38. Smith TO, Postle K, Penny F, McNamara I, Mann CJ. Is reconstruction the best management strategy for anterior cruciate ligament rupture? A systematic review and meta-analysis comparing anterior cruciate ligament reconstruction versus non-operative treatment. *Knee*. 2014;21(2):462-70.
39. Springer BD, Connelly SE, Odum SM, *et al*. Cementless femoral components in young patients: review and meta-analysis of total hip arthroplasty and hip resurfacing. *J Arthroplasty*. 2009;24(6 Suppl):2-8.
40. Thoma A, Veltri K, Haines T, Duku E. A meta-analysis of randomized controlled trials comparing endoscopic and open carpal tunnel decompression. *Plast Reconstr Surg*. 2004;114(5):1137-46.
41. Thorlund JB, Juhl CB, Roos EM, Lohmander LS. Arthroscopic surgery for degenerative knee: systematic review and meta-analysis of benefits and harms. *Br J Sports Med*. 2015;49(19):1229-35.
42. van de Graaf VA, Wolterbeek N, Mutsaerts EL, *et al*. Arthroscopic Partial Meniscectomy or Conservative Treatment for Nonobstructive Meniscal Tears: A Systematic Review and Meta-analysis of Randomized Controlled Trials. *Arthroscopy*. 2016;32(9):1855-65 e4.
43. Vasiliadis HS, Georgoulas P, Shrier I, Salanti G, Scholten RJ. Endoscopic release for carpal tunnel syndrome. *Cochrane Database Syst Rev*. 2014(1):CD008265.
44. Verdugo RJ, Salinas RA, Castillo JL, Cea JG. Surgical versus non-surgical treatment for carpal tunnel syndrome. *Cochrane Database Syst Rev*. 2008(4):CD001552.
45. Wang X, Wanyan P, Tian JH, Hu L. Meta-analysis of randomized trials comparing fusion surgery to non-surgical treatment for discogenic chronic low back pain. *J Back Musculoskelet Rehabil*. 2015;28(4):621-7.
46. Wei J, Song Y, Sun L, Lv C. Comparison of artificial total disc replacement versus fusion for lumbar degenerative disc disease: a meta-analysis of randomized controlled trials. *Int Orthop*. 2013;37(7):1315-25.
47. Xu C, Zhao J. A meta-analysis comparing meniscal repair with meniscectomy in the treatment of meniscal tears: the more meniscus, the better outcome? *Knee Surg Sports Traumatol Arthrosc*. 2015;23(1):164-70.
48. Xu S, Wang J, Liang Y, *et al*. Decompression with fusion is not in superiority to decompression alone in lumbar stenosis based on randomized controlled trials: A PRISMA-compliant meta-analysis. *Medicine (Baltimore)*. 2019;98(46):e17849.
49. Yajun W, Yue Z, Xiuxin H, Cui C. A meta-analysis of artificial total disc replacement versus fusion for lumbar degenerative disc disease. *Eur Spine J*. 2010;19(8):1250-61.
50. Yang LH, Liu W, Li J, *et al*. Lumbar decompression and lumbar interbody fusion in the treatment of lumbar spinal stenosis: A systematic review and meta-analysis. *Medicine (Baltimore)*. 2020;99(27):e20323.
51. Zaina F, Tomkins-Lane C, Carragee E, Negrini S. Surgical versus non-surgical treatment for lumbar spinal stenosis. *Cochrane Database Syst Rev*. 2016(1):CD010264.
52. Zhao XW, Ma JX, Ma XL, *et al*. Interspinous process devices (IPD) alone versus decompression surgery for lumbar spinal stenosis (LSS): A systematic review and meta-analysis of randomized controlled trials. *Int J Surg*. 2017;39:57-64.
53. Zigler J, Gornet MF, Ferko N, Cameron C, Schranck FW, Patel L. Comparison of Lumbar Total Disc Replacement With Surgical Spinal Fusion for the Treatment of Single-Level Degenerative Disc Disease: A Meta-Analysis of 5-Year Outcomes From Randomized Controlled Trials. *Global Spine J*. 2018;8(4):413-23.
54. Zuo D, Zhou Z, Wang H, *et al*. Endoscopic versus open carpal tunnel release for idiopathic carpal tunnel syndrome: a meta-analysis of randomized controlled trials. *J Orthop Surg Res*. 2015;10:12.

Appendix 6. Characteristics of included meta-analyses or relevant studies

**Procedure** First author, year	**Journal title**	**Last search date**	**Databases searched**	**No. of RCTs included**	**Intervention**	**Comparator**	**Condition**	**Key findings**	**GRADE results**
**Arthroscopic anterior cruciate ligament reconstruction**									
Lien-Iversen, 2020	British Journal of Sports Medicine	Oct, 2018	EMBASE, MEDLINE, CINAHL, CENTRAL	1	Minimally invasive ACL reconstruction	Non-surgical treatment	ACL rupture	The risk of radiographic knee OA was higher (RR 1.42; 95% CI 1.09 to 1.85; p=0.009), but the risk of secondary meniscectomy was lower (RR 0.34; 95% CI 0.20 to 0.58; p<0.001) ten years after surgical treatment of ACL rupture. The risk of graft rupture/secondary ACL revision or secondary reconstruction was unrelated to treatment type (RR 0.90; 95% CI 0.49 to 1.66; p=0.74). The degree of knee laxity was reduced after surgical treatment in comparison with non-surgical treatment (p=0.013), while PROMs were similar (KOOS: p=0.35)	Not used
Monk, 2016	Cochrane Database of Systematic Reviews	Jan, 2016	Cochrane Bone Joint and Muscle Trauma Group Specialised Register, CENTRAL, MEDLINE In-Process and Other Non-Indexed Citations, EMBASE, WHO ICTRP, ClinicalTrials.gov	1	ACL reconstruction	Non-surgical treatment	ACL rupture	No difference between surgical management (ACL reconstruction followed by structured rehabilitation) and conservative treatment (structured rehabilitation only) in patient-reported outcomes (KOOS) of knee function at two (MD -0.20; 95% CI -6.78 to 6.38) and five years (MD -2.00; 95% CI -8.27 to 4.27) after injury	Very low to low certainty evidence
Smith, 2014	The Knee	Apr, 2013	AMED, CINAHL, EMBASE, PubMed, psycINFO, MEDLINE, CENTRAL, OpenGrey, WHO ICRTP, Current Controlled Trials, UK National Research Register Archive	1	ACL reconstruction	Non-surgical treatment	ACL rupture	Limited difference in clinical outcomes (KOOS (ADL)) between people managed non-operatively versus with an isolated ACL reconstruction following ACL rupture (MD -1.89; 95% CI -2.24 to -1.55; p<0.001). Lower incidence of partial meniscectomy at longer-term follow-up (ten years and over) (MD 0.18; 95% CI 0.07 to 0.46; p<0.001)	Not used
**Arthroscopic meniscal repair**									
Xu, 2015	Knee Surgery, Sports, Traumatology, Arthroscopy	Mar, 2012	MEDLINE, EMBASE, Ovid	1	Open or arthroscopic meniscal repair	Arthroscopic total or partial meniscectomy	Meniscal tears	Meniscal repairs have better long-term patient-reported outcomes and better activity levels than meniscectomy (IKDC: OR 0.36; 95% CI 0.02 to 6.13; p=0.48; Tenger: MD -0.81; 95% CI -1.13 to -0.49; p<0.001); besides, the former meniscal repairs have a lower failure rate (MD 0.27; 95% CI 0.08 to 0.91; p=0.03)	Not used
**Arthroscopic partial meniscectomy**									
Abram, 2020	British Journal of Sports Medicine	Oct, 2018	MEDLINE, EMBASE, CENTRAL, Scopus, Web of Science, ISRCTN, ClinicalTrials.gov	10	Arthroscopic partial meniscectomy	Non-surgical treatment or sham surgery or placebo or no treatment	Degenerative meniscal tears	Performing APM in all patients with knee pain and a meniscal tear is not appropriate, and surgical treatment should not be considered the first-line intervention. There may, however, be a small-to-moderate benefit from APM compared with physiotherapy for patients without OA (KOOS (pain, 6-12 months): MD 6.91; 95% CI 2.87 to 10.94; p=0.03; KOOS (function, 6-12 months): MD 5.31; 95% CI 1.12 to 9.51; p=0.01)	Very low to high certainty evidence

**Procedure** First author, year	**Journal title**	**Last search date**	**Databases searched**	**No. of RCTs included**	**Intervention**	**Comparator**	**Condition**	**Key findings**	**GRADE results**
Brignardello-Petersen, 2017	BMJ Open	Aug, 2016	MEDLINE, EMBASE, CENTRAL, Google Scholar, OpenGrey	15	Arthroscopic debridement +/- partial meniscectomy	Non-surgical treatment (exercise therapy, injections, drugs, sham surgery)	Symptomatic degenerative knee disease	Over the long term, patients who undergo knee arthroscopy versus those who receive conservative management strategies do not have important benefits in pain (MD 3.1; 95% CI -0.2 to 6.4) or function (MD 3.2; 95% CI -0.5 to 6.8) at two years	Low to high certainty evidence
Khan, 2014	Canadian Medical Association Journal	Jan, 2014	MEDLINE, EMBASE, CENTRAL, PubMed, ClinicalTrials.gov	7	Arthroscopic meniscal debridement	Non-surgical treatment or sham treatments	Degenerative meniscal tears	There is moderate evidence to suggest that there is no benefit to arthroscopic meniscal debridement for degenerative meniscal tears in comparison with nonoperative or sham treatments in middle-aged patients with mild or no concomitant OA (KOOS (function, two years): MD 1.6; 95% CI -2.2 to 5.2); VAS (pain, two years): MD -0.06; 95% CI -0.28 to -0.15)	Not used
Lee, 2018	Archives of Orthopaedic and Trauma Surgery	Aug, 2017	MEDLINE, EMBASE, CENTRAL, Web of Science, Scopus	9	Arthroscopic meniscal debridement	Non-surgical treatment	Degenerative meniscal tears in patients aged ≥40	The efficacy of arthroscopic surgery was not superior to conservative management in this type of patients (pain: SMD 0.01; 95% CI -0.15 to 0.19; p=0.86; function: SMD 0.01; 95% CI -0.19 to 0.21; p=0.93)	Not used
Thorlund, 2015	British Journal of Sports Medicine	Aug, 2014	MEDLINE, EMBASE, CINAHL, Web of Science, CENTRAL	9	Arthroscopic debridement +/- partial meniscectomy	Non-surgical treatment or sham surgery	Degenerative knee disease	There is a small inconsequential benefit (VAS (pain, three months): SMD 0.27; 95% CI 0.14 to 0.41) from interventions that include arthroscopy for the degenerative knee, but this benefit is absent at one to two years after surgery (VAS (pain, two years): SMD 0.06; 95% CI -0.13 to 0.25; VAS (function, two years): SMD -0.02; 95% CI -0.25 to 0.20). Knee arthroscopy is associated with harms. These findings do not support arthroscopic surgery for middle-aged/older patients with knee pain with or without OA	Not used
van de Graaf, 2016	Arthroscopy	May, 2016	CENTRAL, MEDLINE, EMBASE, Physiotherapy Evidence Database, NHS CRD	6	Arthroscopic partial meniscectomy	Non-surgical treatment	Degenerative meniscal tears	Small statistically significant favourable results of APM up to six months for physical function and pain (LKSS (three months): MD 3.31; 95% CI 0.69 to 5.93; p=0.01; WOMAC (function, 6 months): MD 3.56; 95% CI 0.24, 6.88; p=0.04). However, no differences at longer follow-up (WOMAC (function, 12 months): MD 1.14; 95% CI -2.01 to 4.30; p=0.48; LKSS (24 months): MD -1.14; 95% CI -3.72 to 1.45; p=0.39	Very low to high certainty evidence
**Arthroscopic rotator cuff repair**									
Ji, 2015	Arthroscopy	Oct, 2013	PubMed, EMBASE, Scopus, CENTRAL, Cochrane Database of Systematic Reviews	5	Arthroscopic rotator cuff repair	Mini-open rotator cuff repair	Rotator cuff tear	No differences in surgery time (MD 17.64; 95% CI -3.87 to 39.16; p=0.11), function (MD 1.10; 95% CI -3.59 to 5.79); p=0.85), VAS (pain) (MD 0.01; 95% CI -0.21 to 0.22; p=0.96), and range of motion (MD 3.19; 95% CI -1.44 to 7.81; p=0.18) at the end of follow-up between the arthroscopic and mini-open rotator cuff repair techniques. In addition, there was no significant difference in VAS pain score in the early phase between the two repairs (MD -0.10; -1.43 to 1.24; p=0.89)	Not used
Karjalainen, 2019	Cochrane Database of Systematic Reviews	Jan, 2019	CENTRAL, MEDLINE, EMBASE, WHO ICRTP, ClinicalTrials.gov	9	Surgical treatment	Non-surgical treatment (exercises with or without glucocorticoid injection)	Rotator cuff tear (full thickness)	Uncertain whether rotator cuff repair surgery provides clinically meaningful benefits to people with symptomatic tears; it may provide little/no clinically important benefits for pain (MD -0.76; 95% CI -1.20 to -0.32; p<0.001), function (MD 2.83; 95% CI -1.16 to 6.83; p=0.16), overall quality of life or participant-rated global assessment of treatment success (RR 1.06; 95% CI 0.95 to 1.19) when compared with non-operative treatment beyond 12 months. Surgery may not improve shoulder pain or function compared with exercises, with or without glucocorticoid injections	Very low to moderate certainty evidence

**Procedure** First author, year	**Journal title**	**Last search date**	**Databases searched**	**No. of RCTs included**	**Intervention**	**Comparator**	**Condition**	**Key findings**	**GRADE results**
Schemitsch, 2019	The Bone and Joint Journal	Mar, 2018	PubMed, MEDLINE, CENTRAL	6	Surgical treatment (mini-open, open, or arthroscopic)	Non-surgical treatment or subacromial decompression alone	Rotator cuff tear (chronic/ degenerative)	Surgical repair results in significantly improved outcomes when compared with either conservative treatment (Constant-Murley: MD 6.15; 95% CI 2.24 to 10.07; p=0.002) or subacromial decompression alone (Constant-Murley: MD 5.81; 95% CI 2.58 to 9.04; p<0.001) for degenerative rotator cuff tears in older patients	Not used
**Arthroscopic subacromial decompression**									
Karjalainen, 2019	Cochrane Database of Systematic Reviews	Oct, 2018	CENTRAL, MEDLINE, ClinicalTrials.gov, WHO ICRTP	8	Arthroscopic subacromial decompression	Placebo surgery, no intervention or non-surgical intervention	Subacromial impingement syndrome	No clinically important benefit of intervention over placebo in VAS pain (MD -0.36; 95% CI -0.84 to 0.33; p=0.39), function (MD 2.76; 95% CI -1.36 to 6.87; p=0.19), health related QOL (SMD -0.09; 95% CI -0.39 to 0.21; p=0.54) or participant-rated global assessment of treatment success (RR 1.08; 95% CI 0.93 to 1.27; p=0.32) at 12 months	High certainty evidence
Lahdeoja, 2020	British Journal of Sports Medicine	Jul, 2018	MEDLINE, EMBASE, PubMed, CENTRAL, CINAHL, PEDro, ClinicalTrials. Gov, WHO ICRTP	9	Open or arthroscopic subacromial decompression plus postoperative physiotherapy	Placebo surgery or exercise therapy	Subacromial pain syndrome	No clinically important benefit of intervention compared with placebo surgery or exercise therapy in VAS pain (MD -0.26; 95% CI -0.84 to 0.33; p=0.39), Constant function (MD 2.75; 95% CI -1.36 to 6.87; p=0.19) or EQ-5D health related QOL (SMD -0.09; 95% CI -0.39 to 0.21; p=0.54) at 12 months	Moderate to high certainty evidence
Nazari, 2019	Plos One	Nov, 2018	MEDLINE, EMBASE, CINAHL, PubMed	11	Open or arthroscopic subacromial decompression plus postoperative physiotherapy	Placebo surgery plus physiotherapy or physiotherapy only	Subacromial impingement syndrome	No clinically important benefit of intervention plus physiotherapy over physiotherapy in VAS pain (MD 1.00; 95% CI -0.24 to 2.24; p=0.11) and function (SMD 0.22; 95% CI -0.12 to 0.56; p=0.21) at any time point up to ten years	Low to moderate certainty evidence
**Carpal tunnel decompression**									
Chen, 2014	Archives of Orthopaedic and Trauma Surgery	Dec, 2012	CENTRAL, PubMed, EMBASE	15	Open carpal tunnel release	Endoscopic carpal tunnel release	Carpal tunnel syndrome	Endoscopic and open release were similar in relief of symptoms (RR 1.02; 95% CI 0.92 to 1.14; p=0.71) after three months. Endoscopic release resulted in a better recovery of function (grip strength: MD 1.96; 95% CI -0.47 to 4.38; p=0.11; pinch strength: MD 0.93; 95% CI 0.31 to 1.35; p=0.002) and earlier return to work (MD -8.21; 95% CI -9.79 to -6.63; p<0.001) and was determined to be safer than open release	Not used
Hu, 2016	Brain and Behaviour	Jun, 2015	PubMed, EMBASE, MEDLINE, CENTRAL, Association Annual Congress	5	Open carpal tunnel release in one wrist	Endoscopic carpal tunnel release in the other wrist	Bilateral carpal tunnel syndrome	Endoscopic release promoted better recovery of daily life functions (MD 0.13; 95% CI 0.02 to 0.25; p=0.02) but required a longer operative time (MD -1.27; 95% CI -2.22 to -0.33; p=0.008). No difference in VAS pain (MD 0.02; 95% CI -0.08 to 0.11; p=0.75), grip strength (MD 0.17; 95% CI -2.03 to 2.37; p=0.89) and complication rates (RD 0.01; 95% CI -0.02 to 0.05; p=0.47)	Not used
Li, 2019	Medicine (Baltimore)	Jun, 2017	MEDLINE, Web of Science, EMBASE	13	Limited incision carpal tunnel release	Standard incision carpal tunnel release	Carpal tunnel syndrome	Limited incision release allows earlier to return to activities (MD -8.80; 95% CI -9.21 to -8.39; p<0.001), reduces operative time (MD -1.68; 95% CI -3.24 to -0.12; p=0.04), decreases rate of adverse events (RR 0.61; 95% CI 0.38 to 0.96; p=0.03), and improves strength (grip: MD 4.25; 95% CI 0.86 to 7.65; p=0.01; pinch: MD 1.37; 95% CI 0.24 to 2.51; p=0.02) during the early postoperative period. Results at six months or longer are similar according to current data	Not used

**Procedure** First author, year	**Journal title**	**Last search date**	**Databases searched**	**No. of RCTs included**	**Intervention**	**Comparator**	**Condition**	**Key findings**	**GRADE results**
Sanati, 2011	Journal of Occupational Rehabilitation	Dec, 2009	CENTRAL, MEDLINE, AMED, CINAHL	15	Minimally invasive carpal tunnel release	Open carpal tunnel release	Carpal tunnel syndrome	Minimally invasive surgery resulted in earlier return to work (MD -7.22; 95% CI -10.01 to -4.43; p=0.36)	Not used
Sayegh, 2015	Clinical Orthopaedics and Related Research	Apr, 2014	MEDLINE, CINAHL, CENTRAL	21	Open carpal tunnel release	Endoscopic carpal tunnel release	Carpal tunnel syndrome	Endoscopic release allows earlier return to work (MD -8.73; 95% CI -12.82 to -4.65; p<0.001) and improved strength during the early postoperative period but non-significant at six months (grip: MD 0.90; 95% CI -1.47 to 3.27; p=0.46; pinch: MD 0.37; 95% CI -0.09 to 0.84; p=0.12). Results at six months or later are similar according to current data except that patients undergoing endoscopic release are at greater risk of nerve injury (RR 2.84; 95% CI 1.08 to 7.46; p=0.03) and lower risk of scar tenderness (RR 0.53; 95% CI 0.35 to 0.82; p=0.01) compared with open release	Not used
Thoma, 2004	Plastic and Reconstructive Surgery	2002	CENTRAL, MEDLINE, EMBASE, CINAHL, HealthSTAR	13	Open carpal tunnel release	Endoscopic carpal tunnel release	Carpal tunnel syndrome	Endoscopic release is favourable for grip (SMD 0.68; 95% CI 0.06 to 1.30; p=0.01) and pinch (SMD 0.38; 95% CI 0.09 to 0.66; p=0.69) strength at short-term follow-up. Results are inconclusive in terms of pain (OR 3.09; 95% CI 0.69 to 13.80; p<0.001) and return to work (OR 1.52; 95% CI 0.28 to 8.34; p<0.001). Reversible nerve damage was three times as likely to occur with endoscopic release as with open (OR 0.33; 95% CI 0.12 to 0.91; p=0.98)	Not used
Vasiliadis, 2014	Cochrane Database of Systematic Reviews	Nov, 2013	CENTRAL, MEDLINE, EMBASE, Cochrane Neuromuscular Disease Group Trials Register, ClinicalTrial.gov, Current Controlled Trials, Wellcome Trust, UK Clinical Trials Gateway, WHO ICRTP	28	Endoscopic carpal tunnel release	Alternative carpal tunnel release	Carpal tunnel syndrome	Open and endoscopic release for CTS are about as effective as each other in relieving symptoms (SMD -0.13; 95% CI -0.47 to -0.21; p=0.45) and improving functional status (SMD -0.23; 95% CI -0.60 to 0.14; p=0.22), although there may be a functionally significant benefit of endoscopic over open in improvement in grip strength (SMD 0.36; 95% CI 0.09 to 0.63; p=0.008). Endoscopic appears to be associated with fewer minor complications (RR 0.55; 95% CI 0.38 to 0.81; p=0.003) compared to open, but we found no difference in the rates of major complications. Return to work is faster after endoscopic release (MD -4.89; 95% CI -11.35 to 1.57)	Very low to low certainty evidence
Verdugo, 2008	Cochrane Database of Systematic Reviews	Jan, 2008	Cochrane Neuromuscular Disease Group Trials Register, MEDLINE, EMBASE, LILACS	4	Surgical treatment	Non-surgical treatment (splinting or corticosteroid injections)	Carpal tunnel syndrome	Surgical treatment of carpal tunnel syndrome relieves symptoms significantly better than splinting (RR 1.23; 95% CI 1.04 to 1.46). Further research is needed to discover whether this conclusion applies to people with mild symptoms and whether surgical treatment is better than steroid injection	Not used
Zuo, 2015	Journal of Orthopaedic Surgery and Research	Sep, 2013	Google Scholar, MEDLINE, EMBASE, CENTRAL	13	Open carpal tunnel release	Endoscopic carpal tunnel release	Carpal tunnel syndrome	Although ECTR significantly reduced postoperative hand pain (RR 0.70; 95% CI 0.53 to 0.93; p=0.02), it increased the possibility of reversible postoperative nerve injury (RR 2.38; 95% CI 0.98 to 5.77; p=0.05) in patients with idiopathic CTS. No statistical difference in the overall complication rate (RR 1.34; 95% CI 0.74 to 2.43; p=0.34), subjective satisfaction (RR 1.00; 95% CI 0.93 to 1.08; p=0.92), the time to return to work (MD -3.52; 95% CI -8.15 to 1.10; p=0.14), postoperative grip (MD 2.39; 95% CI -0.93 to 5.73; p=0.16) and pinch (MD -0.53; 95% CI -3.16 to 2.11; =0.70) strength, and operative time (MD -4.00, 95% CI -8.01 to 0.00; p=0.05) was observed between the two groups	Not used
**Lumbar spine decompression**									

**Procedure** First author, year	**Journal title**	**Last search date**	**Databases searched**	**No. of RCTs included**	**Intervention**	**Comparator**	**Condition**	**Key findings**	**GRADE results**
Kovacs, 2011	Spine	Jul, 2009	CENTRAL, MEDLINE, EMBASE and TripDatabase	5	Surgical treatment	Non-surgical treatment	Degenerative lumbar spinal stenosis	In patients with symptomatic LSS, the implantation of a specific type of device or decompressive surgery, with or without fusion, is more effective than continued conservative treatment when the latter has failed for three to six months (ODI: MD -10.55; 95% CI -16.88 to -4.22; p=0.001)	Not used
Li, 2017	Neurosurgical Review	Dec, 2014	MEDLINE, PubMed and CENTRAL	8	Interspinous process device implantation	Other treatment options (non-operative therapy, or laminectomy +/- fusion)	Neurogenic intermittent claudication secondary to degenerative spinal stenosis	IPD seems to be more effective than nonoperative treatment and also laminectomy combined with instrumented spinal fusion in treating NIC secondary to spinal stenosis or low-grade degenerative spondylolisthesis; however only limited meta-analysis was performed – more evidence is needed to confirm this. IPD implantation however is not superior to laminectomy and even resulted in a higher reoperation rate (RR 3.75; 95% CI 1.87 to 7.49)	Not used
Ma, 2017	International Journal of Surgery	Sep, 2016	PubMed, CENTRAL, Ovid, MEDLINE, China National Knowledge database, Wanfang database	9	Surgical treatment	Non-surgical treatment	Degenerative lumbar spinal stenosis	Surgery groups showed better late clinical outcomes after one year (ODI (one year): MD -5.89; 95% CI -11.39 to -0.40; p=0.04; ODI (four years): MD -9.40; 95% CI -12.74 to -6.06; p<0.001) and higher complication rate (RR 2.85; 95% CI 1.37 to 5.92; p=0.005) throughout the follow-up duration, although it had no significant differences compared with conservative groups in the first six months post-treatment. However, there was no evidence that a definitive method could be firmly recommended to LSS patients	Not used
Machado, 2016	Cochrane Database of Systematic Reviews	Jun, 2016	Cochrane Back and Neck Review Group Trials Register, CENTRAL, MEDLINE, EMBASE, CINAHL, AMED, Web of Science, LILACS, ClinicalTrials.gov, ANZCTR, WHO ICTRP	24	Surgical treatment	No treatment or placebo or sham surgery	Degenerative lumbar spinal stenosis	There is a paucity of evidence on the efficacy of surgery for lumbar spinal stenosis, as to date no trials have compared surgery with no treatment, placebo or sham surgery. The results demonstrate that at present, decompression plus fusion and interspinous process spacers have not been shown to be superior to conventional decompression alone. Decompression alone versus decompression plus fusion (pain: MD 1.09; 95% CI -4.07 to 6.26; p=0.66). Decompression alone versus interspinous spacers (pain: MD -0.89; 95% CI -6.08 to 4.31; p=0.74)	Very low certainty evidence
Mo, 2018	Pakistan Journal of Medical Sciences	Jun, 2017	PubMed, CENTRAL, Web of Science, Ovid, PEDro	3	Exercise therapy	Surgical treatment	Degenerative lumbar spinal stenosis	Exercise therapy had a similar effect for LSS as decompressive laminectomies (ODI (two years): MD -0.67; 95% CI -6.16 to 4.82; p=0.81; SF-36 (two years): MD 3.85; 95% CI 0.48 to 7.22; p=0.03)	Not used
Overdevest, 2015	Cochrane Database of Systematic Reviews	Jun, 2014	CENTRAL, MEDLINE, MEDLINE In-Process and Other Non-Indexed Citations, EMBASE, Web of Science, WHO ICTRP, ClinicalTrial.gov	10	Posterior decompression (unilateral laminotomy for bilateral decompression, bilateral laminotomy and split-spinous process laminotomy)	Conventional laminectomy	Degenerative lumbar spinal stenosis	Evidence on functional disability, perceived recovery and leg pain is of low or very low quality. Therefore, further research is necessary to establish whether these techniques provide a safe and effective alternative for conventional laminectomy (ODI: MD -1.68; 95% CI -8.50 to 5.13; p=0.63; pain: MD -1.07; 95% CI -2.15 to 0.00; p=0.05; hospital stay: MD 0.10; 95% CI -0.46 to 0.66; p=0.73; complications: OR 1.21; 95% CI 0.20 to 7.16; p=0.83)	Very low to low certainty evidence
Poetscher, 2018	Plos One	Aug, 2017	MEDLINE, PubMed, EMBASE, CENTRAL, Scopus, LILACS	5	Interspinous process device implantation	Non-surgical treatment or decompression surgery	Degenerative lumbar spinal stenosis	IPD implants had significantly higher rates of reoperation (RR 2.05; 95% CI 1.37 to 3.08; p<0.001), with lower cost-effectiveness	Very low to moderate certainty

Procedure First author, year	Journal title	Last search date	Databases searched	No. of RCTs included	Intervention	Comparator	Condition	Key findings	GRADE results
									evidence
Shen, 2018	Pain Physician	Aug, 2016	PubMed, EMBASE, MEDLINE, CENTRAL, Cochrane Library	5	Spinal decompression with fusion	Spinal decompression alone	Degenerative lumbar spinal stenosis	Additional fusion surgery seems unlikely to result in better outcomes (satisfaction: SMD -0.11; 95% CI -0.46 to 0.24; p<=0.53; ODI (two years): SMD 1.64; 95% CI -7.07 to 10.36; p=0.71) for patients with degenerative LSS, but it may increase additional risks in terms of operative duration (SMD -130.37; 95% CI -212.54 to -130.37; p=0.002) and blood loss (SMD -461.78; 95% CI -639.15 to -284.42; p<0.001)	Not used
Xu, 2019	Medicine (Baltimore)	Dec, 2018	PubMed, MEDLINE, EMBASE, CENTRAL, Web of Science	9	Spinal decompression alone	Spinal decompression with fusion	Degenerative lumbar spinal stenosis	Fusion group has no better clinical results than decompression alone in LSS, regardless of degenerative spondylolisthesis and follow-up (pain: MD -0.03; 95% CI -0.83 to 0.76; p=0.94; satisfaction: OR 0.74; 95% CI 0.32 to 1.69; p=0.48; ODI: MD 6.58; 95% CI -5.66 to 18.82; p=0.29)	High certainty evidence
Yang, 2020	Medicine (Baltimore)	Jul, 2019	CENTRAL, PubMed, EMBASE	21	Spinal decompression +/- fusion	Different methods of decompression, fusion or both	Degenerative lumbar spinal stenosis	No overall optimal therapy for lumbar stenosis (ODI: SMD -0.51; 95% CI -1.05 to 0.03; p=0.44)	Not used
Zaina, 2016	Cochrane Database of Systematic Reviews	Feb, 2015	CENTRAL, MEDLINE, MEDLINE In-Process and Other Non-Indexed Citations, EMBASE, CINAHL, Index to Chiropractic Literature, PEDro, ClinicalTrial.gov, WHO ICTRP, PubMed, Cochrane Back and Neck Review Group Trials Register	5	Surgical treatment	Non-surgical treatment	Degenerative lumbar spinal stenosis	No clear benefits were observed with surgery versus non-surgical treatment (ODI (one year): MD -6.17; 95% CI -15.02 to 2.67; p=0.17)	Low certainty evidence
Zhao, 2017	International Journal of Surgery	Aug, 2016	PubMed, Cochrane Library, CENTRAL, MEDLINE, China National Knowledge database, Wanfang database	4	Interspinous process device implantation	Spinal decompression	Degenerative lumbar spinal stenosis	Although patients who received IPD may obtain several benefits in the short term (VAS pain: MD 9.65; 95% CI 0.78 to 18.51; p=0.03), it was associated with higher costs and reoperation rates (RR 2.91; 95% CI 1.72 to 4.92; p<0.001). Both IPD and bony decompression were acceptable strategies for LSS, but the risks, indications, and costs of IPD should be carefully taken into account before surgery	Not used
**Lumbar spine fusion**									
Bai, 2019	Medicine (Baltimore)	Oct, 2018	PubMed, Web of Science, EMBASE, CENTRAL, Chinese Knowledge Infrastructure database, Wanfang database, VIP database	14	Total disc replacement	Lumbar fusion	Lumbar degenerative disc disease	TDR is recommended to alleviate the pain (VAS: SMD -0.21; 95% CI -0.33 to -0.09; p=0.001) of degenerative lumbar diseases, improve the state of lumbar function (ODI: SMD -0.28; 95% CI -0.40 to -0.15; p<0.001) and the quality of life (SF-36: SMD 0.28; 95% CI 0.16 to 0.41; p<0.001) of patients, provide a high level of security, have better health economics benefits for one-level patients	Low to moderate certainty evidence

**Procedure** First author, year	**Journal title**	**Last search date**	**Databases searched**	**No. of RCTs included**	**Intervention**	**Comparator**	**Condition**	**Key findings**	**GRADE results**
Bydon, 2014	Journal of Spinal Disorders and Techniques	Aug, 2013	PubMed and CENTRAL	5	Lumbar fusion	Non-surgical treatment (physical therapy, patient education, exercise, pain relief by acupuncture and injections)	Chronic discogenic lower back pain	No significant difference when compared with the nonoperative group (ODI: MD -7.39; 95% CI -20.26 to 5.47; p=0.26)	Not used
Hiratzka, 2015	Global Spine Journal	May, 2015	PubMed, CENTRAL, National Guideline Clearinghouse database	7	Lumbar fusion	Total disc replacement	Lumbar degenerative disc disease	Lumbar TDR appears to be comparable in safety to lumbar fusion over five years (adverse events: RR 1.86; 95% CI 0.68 to 5.10; p=0.23; reoperation: RR 1.10; 95% CI 0.56 to 2.15; p=0.78)	Not used
Jacobs, 2012	Cochrane Database of Systematic Reviews	Dec, 2011	CENTRAL, CBRG trials register, MEDLINE, EMBASE, BIOSIS, FDA register and ClinicalTrials.gov	6	Total disc replacement	Lumbar spine fusion	Lumbar degenerative disc disease	Although statistically significant, the differences between disc replacement and conventional fusion surgery for degenerative disc disease were not beyond the generally accepted clinical important differences for short-term pain relief (VAS: MD 5.22; 95% CI 0.18 to 10.26; p=0.04), disability (ODI: OR 1.45; 95% CI 1.08 to 1.98; p=0.02) and QOL. Moreover, these analyses only represent a highly selected population	Very low to moderate certainty evidence
Li, 2018	Journal of Orthopaedic Surgery and Research	Feb, 2018	EMBASE, PubMed, Web of Science and Google	7	Open TLIF fusion	Minimally invasive TLIF fusion	Single-level degenerative lumbar disease	MI-TLIF showed significantly less blood loss (SMD -291.46; 95% CI -366.66 to -216.47; p<0.001) compared with O-TLIF and more fluoroscopic time (SMD 35.79; 95% CI 23.31 to 48.27; p<0.001). There was no significant difference between the length of hospital stay (SMD -1.63; 95% CI -3.76 to 0.49; p=0.13), postoperative VAS (SMD -0.19; 95% CI -0.63 to 0.25; p=0.39) or ODI (SMD 0.20; 95% CI -1.18 to 1.58; p=0.78)	Not used
Li, 2020	Turkish Neurosurgery	Jun, 2017	PubMed, EMBASE, CENTRAL	7	Total disc replacement	Lumbar fusion	Lumbar degenerative disc disease	TDR could be an alternative treatment for LDDD, since it yielded better clinical success (RR 1.10; 95% CI 1.03 to 1.17; p=0.003) and patient satisfaction (RR 1.18; 95% CI 1.10 to 1.27; p<0.001), shorter hospital stays (SMD -0.95; 95% CI -1.55 to -0.35; p=0.002) and operative time (SMD -1.16; 95% CI -1.98 to -0.35; p=0.005), less pain (VAS: SMD -0.18; 95% CI -0.29 to -0.08; p=0.001), and lower complication rates (RR 0.59; 95% CI 0.47 to 0.75; p<0.001) than lumbar fusion	Not used
Miller, 2020	World Neurosurgery	Feb, 2019	MEDLINE, EMBASE, CENTRAL, DOAJ and Google Scholar	7	Open TLIF fusion	Minimally invasive TLIF fusion	Lumbar degenerative disc disease	MI-TLIF is associated with less blood loss (MD -200; 95% CI -307 to -93; p<0.001), shorter hospital stays (MD -2.2; 95% CI -2.7 to -1.7; p<0.001), and slightly less disability (ODI: MD -3; 95% CI -5 to -12; p=0.01), at the expense of longer fluoroscopy time (MD 48; 95% CI 44 to 53; p<0.001)	Not used
Nie, 2015	Journal of the College of Physicians and Surgeons Pakistan	Sep, 2011	PubMed, EMBASE and CENTRAL	6	Total disc replacement	Lumbar fusion	Lumbar degenerative disc disease	Over a long-term of follow-up (two years) TDR shows a significant superiority for the treatment of LDDD compared with fusion (ODI: MD -4.87; 95% CI -7.77 to -1.97; p=0.001; VAS (pain): MD -5.13; 95% CI -9.02 to -1.25; p=0.01; satisfaction: OR 1.68; 95% CI 1.26 to 2.25; p<0.001; complications: OR 0.65; 95% CI 0.29 to 0.84; p=0.08)	Not used
Rao, 2014	Archives of Orthopaedic and Trauma Surgery	Mar, 2013	MEDLINE, EMBASE, Clinical, Ovid, BIOSIS, CENTRAL, Spine, European Spine Journal, Journal of	7	Total disc replacement	Lumbar fusion	Lumbar degenerative disc disease	TDR showed significant safety and efficacy (ODI: MD -5.09; 95% CI -7.33 to -2.64; p<0.001) comparable to lumbar fusion at two-year follow-up. TDR demonstrated superiorities in improved physical function, reduced pain (VAS: MD -5.31; 95% CI -8.35 to 2.28; p<0.001) and shorten duration of hospitalisation (MD -0.82; 95% CI -1.38 to -0.26; p=0.004)	Not used

**Procedure** First author, year	**Journal title**	**Last search date**	**Databases searched**	**No. of RCTs included**	**Intervention**	**Comparator**	**Condition**	**Key findings**	**GRADE results**
			Bone and Joint Surgery (US) and Journal of Bone and Joint Surgery (UK)						
Wang, 2015	Journal of Back and Musculoskeletal Rehabilitation	Mar, 2013	PubMed, CENTRAL, EMBASE, Science Citation Index and Chinese Biomedial Literature database	6	Lumbar fusion	Non-surgical treatment (physical exercises and cognitive therapy)	Chronic discogenic lower back pain	Fusion surgery was not superior to nonsurgical treatment in terms of changes in ODI (MD 1.94; 95% CI -6.02 to 2.14) scores for DLBP. Fusion surgery resulted in surgical complications (RR 22.11; 95% CI 55.99 to 81.60)	Not used
Wei, 2013	International Orthopaedics	Jan, 2013	PubMedCentral, MEDLINE, EMBASE, BIOSIS, ClinicalTrials.gov and FDA trials register	6	Total disc replacement	Lumbar fusion	Lumbar degenerative disc disease	TDR has significant safety and efficacy comparable to lumbar fusion at a two-year follow-up. Although superiority compared to fusion could not be proved, by comparing clinical symptoms relieved (VAS (pain): MD -3.18; 95% CI -5.74 to -0.63; p=0.01; ODI: MD -5.13; 95% CI -7.35 to -2.90; p<0.001), motion preserved, and the low reoperation rate (OR 0.91; 95% CI 0.57 to 1.46; p=0.71) during long-term follow-up on TDR, TDR was considered safe and effective	Not used
Yajun, 2010	European Spine Journal	Jul, 2009	PubMed, CENTRAL, Ovid, MEDLINE, EMBASE, Spine, ropean Spine Journal an, Journal of Bone and Joint Surgery	5	Total disc replacement	Lumbar fusion	Lumbar degenerative disc disease	TDR results in a slightly better functioning (ODI: MD -3.92; 95% CI -7.92 to 0.08; p=0.05) and pain (VAS: MD -4.19; 95% CI -9.72 to 1.33) status without clinical significance, and a significantly greater patient satisfaction (SMD 0.29; 95% CI 0.05 to 0.53; p=0.02) at the two-year follow-up point. Omitting the study that used stand-alone cage interbody fusion as the control, there is no longer a significant difference in function and pain status and patient satisfaction between TDR and the fusion group. At five years, these outcomes are not significantly different between comparing groups. Complication (OR 0.64; 95% CI 0.32 to 1.32; p=0.23) and reoperation rate (OR 0.88; 95% CI 0.38 to 2.00; p=0.75) are similar between the two groups at two and five years respectively. From the existing outcomes, the TDR does not show significant superiority for the treatment of lumbar DDD compared with fusion	Not used
Zigler, 2018	Global Spine Journal	2015	PubMed, MEDLINE and CENTRAL	4	Total disc replacement	Lumbar fusion	Single-level degenerative lumbar disease	TDR is an effective treatment compared with spinal fusion in lumbar DDD. It offers several clinical advantages that can benefit the patient, without the addition of safety consequences (ODI: RR 1.09; 95% CI 1.00 to 1.19; p=0.05; VAS (pain): MD -2.79; 95% CI -8.09 to 2.51; p=0.30; satisfaction: RR 1.13; 95% CI 1.03 to 1.24; p=0.009; reoperation: RR 0.52; 95% CI 0.35 to 0.77; p=0.001)	Not used
**Total hip replacement**									
Jiang, 2011	The Journal of Arthroplasty	Jun, 2009	Cochrane Bone Joint and Muscle Trauma Group Specialised Register, CENTRAL, PubMed, Ovid, ScienceDirect Online	4	Metal-on-metal hip resurfacing arthroplasty	Standard total hip arthroplasty	Hip disease in young active patients	Insufficient evidence to determine whether modern MMHRA offers clinical advantages over standard THR for the treatment of hip disease in active young patients (revision risk: RR 2.60; 95% CI 1.31 to 5.15; p=0.006; dislocation risk: RR 0.25; 95% CI 0.05 to 1.21; p=0.08; three-year mortality: RR 1.05; 95% CI 0.24 to 4.66; p=0.07; loosening risk: RR 4.96; 95% CI 1.82 to 13.50; p=0.002; PJI risk: RR 2.25; 95% CI 0.61 to 8.31; p=0.22; HHS: MD 0.50; 95% CI -0.41 to 1.41; p=0.28)	Not used

**Procedure** First author, year	**Journal title**	**Last search date**	**Databases searched**	**No. of RCTs included**	**Intervention**	**Comparator**	**Condition**	**Key findings**	**GRADE results**
Smith, 2010	Acta Orthopaedica	Jan, 2010	MEDLINE, CINAHL, AMED, EMBASE, SIGLE, National Technical Information Service, National Research Register UK, British Library Integrated Catalogue, Current Controlled Trials	10	Conventional THR	Hip resurfacing	Hip pathology	Resurfacing may have better functional outcomes (WOMAC: MD -2.41; 95% CI -3.88 to -0.94; p=0.001) than THR, but the increased risks of heterotopic ossification (RR 1.62; 95% CI 1.23 to 2.14; p<0.001), aseptic loosening (RR 3.07; 95% CI 1.11 to 8.50; p=0.03), and revision surgery (RR 1.72; 95% CI 1.20 to 2.45; p=0.003) following resurfacing indicate that THA is superior in terms of implant survival	Not used
Springer, 2009	The Journal of Arthroplasty	Mar, 2008	MEDLINE, PubMed, CINAHL	0	THR	Hip resurfacing	Hip pathology	The pooled failure rate for THR was 1.3% (95% CI 1.0 to 1.7), compared to 2.6% (95% CI 2.0 to 3.4) for hip resurfacing, therefore the enthusiasm for hip resurfacing should be tempered by these data	Not used
**Total knee replacement**									
Skou, 2015	New England Journal of Medicine	NA	NA	1	TKR followed by non-surgical treatment	Non-surgical treatment (consisted of exercise, education, dietary advice, use of insoles, and pain medication)	Moderate-to-severe knee OA	Treatment with TKR followed by nonsurgical treatment resulted in greater pain relief (MD 17.1; 95% CI 10.4 to 23.8) and functional improvement (KOOS: MD 15.8; 95% CI 10.0 to 21.5) after 12 months than did nonsurgical treatment alone. However, TKR was associated with a higher number of serious adverse events than was nonsurgical treatment (24 vs 6 months; p=0.005)	NA

Key (alphabetical)
ACL=anterior cruciate ligament; ADL=activities of daily living; AMED=Allied and Complementary Medicine Database; ANZCTR=Australian New Zealand Clinical Trials Registry; APM=arthroscopic partial meniscectomy; CENTRAL=Cochrane Central Register of Controlled Trials; CI=confidence interval; CINAHL=Cumulative Index to Nursing and Allied Health Literature; CTS=carpal tunnel syndrome; DLBP=discogenic lower back pain; ECTR=endoscopic carpal tunnel release; EMBASE=Excerpta Medica Database; EQ-5D=EuroQol health-related quality of life measure; IKDC=International Knee Documentation Committee subjective knee form; HHS=Harris Hip Score; IPD=interspinous process device; ISRCTN=International Standard Randomised, Controlled Trial Number; KOOS=Knee injury and Osteoarthritis Outcome Score; LDDD= lumbar degenerative disc disease; LILACS=Latin American and Caribbean Health Sciences Literature; LKSS=Lysholm Knee Scoring Scale; LSS=lumbar spine stenosis; MD=mean difference; MEDLINE=Medical Literature Analysis and Retrieval System Online; MI-TLIF=minimally invasive transforaminal interbody fusion; MMHRA=metal-on-metal hip resurfacing arthroplasty; NA=Not applicable; NIC=neurogenic intermittent claudication; NHS CRD=National Health Service Centre for Reviews and Dissemination; PJI=periprosthetic joint infection; OA=osteoarthritis; ODI=Oswestry Disability Index; OR=odds ratio; O-TLIF=open transforaminal interbody fusion; PEDro=Physiotherapy Evidence Database; PROM=patient reported outcome measure; QOL=quality of life; RR=relative risk (or risk ratio); SF-36=36-item Short Form survey; SIGLE=System for Information on Grey Literature in Europe; SMD=standardised mean difference; TDR=total disc replacement; THR=total hip replacement; TKR=total knee replacement; TLIF= transforaminal interbody fusion; WHO ICRTP=World Health Organisation International Clinical Trials Registry Platform; WOMAC=Western Ontario and McMaster universities osteoarthritis index; VAS=Visual Analogue Scale.

Appendix 7. AMSTAR scores for the included studies

**Procedure** First author, year	**Was an *a priori* design provided?**	**Was there duplicate study selection and data extraction?**	**Was a comprehensive literature search performed?**	**Was the status of publication (i.e. grey literature) used as an inclusion criterion?**	**Was a list of studies (included and excluded) provided?**	**Were the characteristics of the included studies provided?**	**Was the scientific quality of the included studies assessed and documented?**	**Was the scientific quality of the included studies used appropriately in formulating conclusions?**	**Were the methods used to combine the findings of studies appropriate?**	**Was the likelihood of publication bias assessed?**	**Was the conflict of interest stated?**	**Total score (max. 11)**
**Arthroscopic anterior cruciate ligament reconstruction**												
Lien-Iversen, 2020	1	1	1	1	0	1	1	1	1	0	1	9
Monk, 2016	1	1	1	1	1	1	1	1	1	1	1	11
Smith, 2014	0	1	1	1	1	1	1	1	1	0	1	9
**Arthroscopic meniscal repair**												
Xu, 2015	0	1	1	1	0	1	0	0	1	0	0	5
**Arthroscopic partial meniscectomy**												
Abram, 2020	1	1	1	1	1	1	1	1	1	1	1	11
Brignardello-Petersen, 2017	1	1	1	1	1	1	1	1	1	1	1	11
Khan, 2014	0	1	1	1	0	1	1	1	1	1	1	9
Lee, 2018	0	1	1	1	0	1	1	1	1	0	1	8
Thorlund, 2015	1	1	1	1	1	1	1	1	1	0	1	10
van de Graaf, 2016	1	1	1	1	0	1	1	1	1	1	1	10
**Arthroscopic rotator cuff repair**												
Ji, 2015	0	1	1	1	0	1	1	1	1	0	1	8
Karjalainen, 2019	1	1	1	1	1	1	1	1	1	1	1	11
Schemitsch, 2019	0	1	1	1	0	1	1	1	1	0	0	7
**Arthroscopic subacromial decompression**												
Karjalainen, 2019	1	1	1	1	1	1	1	1	1	1	1	11
Lahdeoja, 2020	1	1	1	1	0	1	1	1	1	1	1	10
Nazari, 2019	1	1	1	1	0	1	1	1	1	0	1	9
**Carpal tunnel decompression**												
Chen, 2014	0	1	1	1	0	1	1	1	1	1	0	8
Hu, 2016	0	1	1	1	0	1	0	0	1	1	1	7
Li, 2019	0	1	1	1	0	1	1	1	1	1	1	9
Sanati, 2011	0	1	1	1	0	1	1	1	1	0	1	8
Sayegh, 2014	0	1	1	1	0	1	1	1	1	1	1	9
Thoma, 2004	0	1	1	1	0	0	1	1	1	1	0	7
Vasiliadis, 2014	1	1	1	1	1	1	1	1	1	1	1	11
Verdugo, 2008	1	1	1	1	1	1	1	1	1	0	1	10
Zuo, 2015	0	1	1	1	1	1	1	1	1	0	1	9

**Procedure** First author, year	**Was an *a priori* design provided?**	**Was there duplicate study selection and data extraction?**	**Was a comprehensive literature search performed?**	**Was the status of publication (i.e. grey literature) used as an inclusion criterion?**	**Was a list of studies (included and excluded) provided?**	**Were the characteristics of the included studies provided?**	**Was the scientific quality of the included studies assessed and documented?**	**Was the scientific quality of the included studies used appropriately in formulating conclusions?**	**Were the methods used to combine the findings of studies appropriate?**	**Was the likelihood of publication bias assessed?**	**Was the conflict of interest stated?**	**Total score (max. 11)**
**Lumbar spine decompression**												
Kovacs, 2011	0	1	1	1	1	1	1	1	1	0	0	8
Li, 2017	0	1	1	1	1	1	1	1	1	0	1	9
Ma, 2017	0	1	1	1	1	1	1	1	1	0	1	9
Machado, 2016	1	1	1	1	1	1	1	1	1	1	1	11
Mo, 2018	0	1	1	1	0	1	1	1	1	0	0	7
Overdevest, 2015	1	1	1	1	1	1	1	1	1	1	1	11
Poetscher, 2018	0	1	1	1	1	1	1	1	1	0	1	9
Shen, 2018	0	1	1	1	0	1	1	1	1	0	1	8
Xu, 2019	1	1	1	1	0	1	1	1	1	1	1	10
Yang, 2020	0	1	1	1	0	1	0	0	1	0	1	6
Zaina, 2016	1	1	1	1	1	1	1	1	1	1	1	11
Zhao, 2017	0	1	1	1	0	1	1	1	1	0	0	7
**Lumbar spine fusion**												
Bai, 2019	1	1	1	1	0	1	1	1	1	1	1	10
Bydon, 2014	0	1	1	1	0	1	1	1	1	0	1	8
Hiratzka, 2015	0	0	1	1	1	1	0	0	1	0	1	6
Jacobs, 2012	1	1	1	1	1	1	1	1	1	1	1	11
Li, 2018	0	1	1	1	0	1	1	1	1	1	1	9
Li, 2020	0	1	1	1	0	1	1	1	1	1	0	8
Miller, 2020	1	1	1	1	0	1	1	1	1	1	1	10
Nie, 2015	0	1	1	1	0	1	1	1	1	1	0	8
Rao, 2014	0	1	1	1	0	1	1	1	1	1	1	9
Wang, 2015	0	1	1	1	0	1	1	1	1	0	0	7
Wei, 2013	0	1	1	1	0	1	1	1	1	0	0	7
Yajun, 2010	0	1	1	1	0	1	1	1	1	1	1	9
Zigler, 2018	0	1	1	1	0	1	1	1	1	0	1	8
**Total hip replacement**												
Jiang, 2011	0	1	1	1	0	0	0	0	1	0	0	4
Smith, 2010	0	1	1	1	0	1	0	0	1	1	1	7
Springer, 2009	0	1	1	1	0	1	0	0	1	0	1	6

Appendix 8. Findings of randomised controlled trials at low risk of bias

First author	Population	Intervention	Comparators	Findings
**Arthroscopic anterior cruciate ligament reconstruction**				
Frobell, 2010 and 2013	121 adults with acute ACL injuries	Structured rehabilitation plus early ACL reconstruction	Structured rehabilitation plus optional later ACL reconstruction	**Two-year outcomes:** No difference in KOOS-4 score (MD 0.2, 95% CI -6.5 to 6.8); No difference in knee-related outcomes, health status, and return to preinjury activity level; adverse events were common in both groups.<br><br>**Five-year outcomes:** No difference in KOOS-4 score (MD -2.00, 95% CI -8.27 to 4.27); treatment failure (RR 0.88, 95% CI 0.62 to 1.25); no difference in Tegner activity score (RR 1.22, 95% CI 0.78 to 1.91); osteoarthritis at five years (RR 1.80, 95% CI 0.92 to 3.52)
**Arthroscopic meniscal repair**				
Biedert, 2000	40 patients with an isolated and symptomatic painful horizontal grade 2 meniscal lesion on the medial side	Arthroscopic repair/arthroscopic minimal resection and repair/arthroscopic partial meniscectomy	Non-surgical treatment	According to IKDC protocols, clinical findings suggested that non-surgical treatment was not satisfactory
**Arthroscopic partial meniscectomy**				
Katz, 2013	Symptomatic patients 45 years of age or older with a meniscal tear and evidence of mild-to-moderate osteoarthritis on imaging	Arthroscopic partial meniscectomy and postoperative physical therapy	Standardised physical-therapy regimen	No significant differences between the study groups in functional improvement WOMAC score (MD 2.4, 95% CI -1.8 to 6.5). The frequency of adverse events was similar between groups
Kise, 2016	140 middle-aged patients with degenerative meniscal tears.	Arthroscopic partial meniscectomy alone	12-week supervised exercise therapy alone	No clinically relevant difference between the two groups in change in KOOS-4 at two years (MD 0.9, 95% CI −4.3 to 6.1). At three months, muscle strength had improved in the exercise group. No serious adverse events occurred in either group.

**Arthroscopic rotator cuff repair**				
Moosmayer, 2010, 2014	103 patients with symptomatic small and medium-sized tears of the rotator cuff	Rotator cuff repair, decompression and exercises	Physiotherapy	At one-year, the between-group differences showed better results for the surgery group on the Constant score (MD 13.0 points), on the ASES scale (MD 16.1 points), for pain-free abduction (MD 28.8°) and reduction in pain (MD on a VAS -1.7 cm). At a five-year follow-up, the results of the surgical group were superior in terms of Constant score, ASES scale, VAS for pain and patient satisfaction. The authors noted that though a surgical repair was associated with a better outcome than physiotherapy treatment, the differences were small and may be below clinical importance.
Kukkonen, 2014, 2015	180 shoulders (173 patients) with supraspinatus tendon tears	Rotator cuff repair, acromioplasty and physiotherapy	Physiotherapy (group 1); Acromioplasty and physiotherapy (group 2)	At one-year, the mean change in the Constant score was 17.0, 17.5, and 19.8, respectively (p=0.34); subscores concerning the range of movement and strength were not significantly different between the groups (p=0.74 and p=0.76, respectively). Patient satisfaction was also not different (p=0.14). The authors concluded that at one-year follow-up, operative treatment is no better than conservative treatment. There was no significant difference in clinical outcome between the interventions at the two-year follow-up.
**Arthroscopic subacromial decompression**				
Beard, 2018	313 patients who had subacromial pain for at least 3 months with intact rotator cuff tendons, were eligible for arthroscopic surgery and had previously completed a non-operative management programme that included exercise therapy and at least one steroid injection	Arthroscopic subacromial decompression	Placebo surgery (group 1); No intervention (group 2)	Mean Oxford Shoulder Score did not differ between the two surgical groups at 6 months (decompression vs arthroscopy (MD -1.3 points, 95% CI –3.9 to 1.3). Both surgical groups showed a small benefit over no treatment, but these differences were not clinically important. There were no differences in complications between the groups.
Paavola, 2018	210 patients with symptoms consistent with shoulder impingement syndrome	Arthroscopic subacromial decompression	Placebo surgery (group 1); Exercise therapy (group 2)	No clinically relevant between-group differences were seen in shoulder pain at rest and on arm activity outcomes at 24 months. No between-group differences were seen between the ASD and diagnostic arthroscopy groups in the secondary outcomes (Constant score, Simple shoulder test score, 15D score, patient satisfaction) or adverse events. The authors concluded that "In this controlled trial involving patients with a shoulder impingement syndrome, arthroscopic subacromial decompression provided no benefit over

				diagnostic arthroscopy at 24 months.
**Carpal tunnel decompression**				
Gerritsen, 2002	176 patients with clinically and electrophysiologically confirmed idiopathic CTS	Open carpal tunnel release surgery	Wrist splinting	Surgery was more effective than splinting on all outcome measures (success rates, number of nights waking up due to symptoms, the severity of the main complaint, paraesthesia during the day and at night. Success rates (based on general improvement) (Difference of 26%, 95% CI 12% to 40%)
Hui, 2005	50 patients with electrophysiologically confirmed idiopathic CTS	Open carpal tunnel release surgery	A single injection of steroid	20 weeks after randomization, patients who underwent surgery had greater symptomatic improvement than those who were injected. There was greater improvement in the Global Symptom Score, median nerve distal motor latencies and sensory nerve conduction velocity. However, the mean grip strength in the surgical group was reduced by 1.7 kg (SD 5.1) compared with a gain of 2.4 kg (SD 5.5) in the injection group.
Jarvik, 2009	116 patients with CTS without denervation	Carpal tunnel surgery	Non-surgical treatment (including hand therapy and ultrasound)	There was a significant 12-month adjusted advantage for surgery in function (CTSAQ function score: Delta -0.40, 95% CI 0.11 to 0.70, p=0.008) and symptoms (CTSAQ symptom score: 0.34, 0.02 to 0.65, p=0.04). There were no clinically important adverse events and no surgical complications.
**Lumbar spine decompression**				
Malmivaara, 2007	94 patients with lumbar spinal stenosis	Segmental decompression and an undercutting facetectomy of the affected area performed	Conservative treatment (NSAIDs and physiotherapy)	Both treatment groups showed improvement during follow-up. At one-year, the mean difference in favour of surgery was 11.3 in disability (95% CI 4.3 to 18.4), 1.7 in leg pain (95% CI 0.4 to 3.0), and 2.3 (95% CI 1.1 to 3.6) in back pain. Walking ability, either reported or measured, did not differ between the two treatment groups.

**Lumbar spine fusion**				
Brox, 2006	60 patients with low back pain lasting longer than 1 year after previous surgery for disc herniation	Lumbar fusion with posterior transpedicular screws	Cognitive intervention and exercises	Oswestry Disability Index was significantly improved from 47 to 38 after fusion and from 45 to 32 after cognitive intervention and exercises (MD -7.3, 95% CI -17.3 to 2.7, p=0.15). The success rate was 50% in the fusion group and 48% in the cognitive intervention/exercise group. For patients with chronic low back pain after previous surgery for disc herniation, lumbar fusion failed to show any benefit over cognitive intervention and exercises.
Brox, 2010	124 patients with disc degeneration and at least 1 year of symptoms after or without previous surgery for disc herniation	Lumbar fusion with posterior transpedicular screws	Cognitive intervention and exercises	At four years, the mean treatment effect for Oswestry Disability Index was 1.1; 95% CI 5.9 to 8.2. There was no difference in return to work.
**Total knee replacement**				
Skou, 2015	100 patients with moderate-to-severe knee osteoarthritis were eligible for unilateral TKR.	TKR followed by non-surgical treatment	Non-surgical treatment (consisted of exercise, education, dietary advice, use of insoles, and pain medication)	The total knee replacement group had greater improvement in the KOOS4 score than did the nonsurgical- treatment group (32.5 vs. 16.0; adjusted mean difference, 15.8 [95% CI 10.0 to 21.5]). The total-knee-replacement group had a higher number of serious adverse events than did the nonsurgical-treatment group (24 vs. 6 months, p=0.005).

ACL, anterior cruciate ligament; ASES, American Shoulder and Elbow Surgeons; CI, confidence interval; CTS, carpal tunnel syndrome; CTSAQ, Carpal Tunnel Syndrome Assessment Questionnaire; IKDC, International Knee Documentation Committee; KOOS, Knee Injury and Osteoarthritis Outcome Score; MD, mean difference; NSAIDs, Non-steroidal anti-inflammatory drugs; RR, relative risk; SD, standard deviation; TKR, total knee replacement; WOMAC, Western Ontario and McMaster Universities Arthritis Index

Appendix 9. Reference list of randomised controlled trials at low risk of bias

1. Beard DJ, Rees JL, Cook JA, Rombach I, Cooper C, Merritt N, Shirkey BA, Donovan JL, Gwilym S, Savulescu J *et al*: Arthroscopic subacromial decompression for subacromial shoulder pain (CSAW): a multicentre, pragmatic, parallel group, placebo-controlled, three-group, randomised surgical trial. *Lancet* 2018, 391(10118):329-338.
2. Biedert RM: Treatment of intrasubstance meniscal lesions: a randomized prospective study of four different methods. *Knee Surg Sports Traumatol Arthrosc* 2000, 8(2):104-108.
3. Brox JI, Nygaard OP, Holm I, Keller A, Ingebrigtsen T, Reikeras O: Four-year follow-up of surgical versus non-surgical therapy for chronic low back pain. *Ann Rheum Dis* 2010, 69(9):1643-1648.
4. Brox JI, Reikeras O, Nygaard O, Sorensen R, Indahl A, Holm I, Keller A, Ingebrigtsen T, Grundnes O, Lange JE *et al*: Lumbar instrumented fusion compared with cognitive intervention and exercises in patients with chronic back pain after previous surgery for disc herniation: a prospective randomized controlled study. *Pain* 2006, 122(1-2):145-155.
5. Frobell RB, Roos HP, Roos EM, Roemer FW, Ranstam J, Lohmander LS: Treatment for acute anterior cruciate ligament tear: five year outcome of randomised trial. *BMJ* 2013, 346:f232.
6. Frobell RB, Roos EM, Roos HP, Ranstam J, Lohmander LS. A randomized trial of treatment for acute anterior cruciate ligament tears. N Engl J Med. 2010 Jul 22;363(4):331-42
7. Gerritsen AA, de Vet HC, Scholten RJ, Bertelsmann FW, de Krom MC, Bouter LM: Splinting vs surgery in the treatment of carpal tunnel syndrome: a randomized controlled trial. *JAMA* 2002, 288(10):1245-1251.
8. Hui AC, Wong S, Leung CH, Tong P, Mok V, Poon D, Li-Tsang CW, Wong LK, Boet R: A randomized controlled trial of surgery vs steroid injection for carpal tunnel syndrome. *Neurology* 2005, 64(12):2074-2078.
9. Jarvik JG, Comstock BA, Kliot M, Turner JA, Chan L, Heagerty PJ, Hollingworth W, Kerrigan CL, Deyo RA: Surgery versus non-surgical therapy for carpal tunnel syndrome: a randomised parallel-group trial. *Lancet* 2009, 374(9695):1074-1081.
10. Katz JN, Brophy RH, Chaisson CE, de Chaves L, Cole BJ, Dahm DL, Donnell-Fink LA, Guermazi A, Haas AK, Jones MH *et al*: Surgery versus physical therapy for a meniscal tear and osteoarthritis. *N Engl J Med* 2013, 368(18):1675-1684.
11. Kise NJ, Risberg MA, Stensrud S, Ranstam J, Engebretsen L, Roos EM: Exercise therapy versus arthroscopic partial meniscectomy for degenerative meniscal tear in middle aged patients: randomised controlled trial with two year follow-up. *BMJ* 2016, 354:i3740.
12. Kukkonen J, Joukainen A, Lehtinen J, Mattila KT, Tuominen EK, Kauko T, Aarimaa V: Treatment of non-traumatic rotator cuff tears: A randomised controlled trial with one-year clinical results. *Bone Joint J* 2014, 96-B(1):75-81.
13. Kukkonen J, Joukainen A, Lehtinen J, Mattila KT, Tuominen EK, Kauko T, Aarimaa V: Treatment of Nontraumatic Rotator Cuff Tears: A Randomized Controlled Trial with Two Years of Clinical and Imaging Follow-up. *J Bone Joint Surg Am* 2015, 97(21):1729-1737.
14. Malmivaara A, Slatis P, Heliovaara M, Sainio P, Kinnunen H, Kankare J, Dalin-Hirvonen N, Seitsalo S, Herno A, Kortekangas P *et al*: Surgical or nonoperative treatment for lumbar spinal stenosis? A randomized controlled trial. *Spine (Phila Pa 1976)* 2007, 32(1):1-8.
15. Moosmayer S, Lund G, Seljom U, Svege I, Hennig T, Tariq R, Smith HJ: Comparison between surgery and physiotherapy in the treatment of small and medium-sized tears of the rotator cuff: A randomised controlled study of 103 patients with one-year follow-up. *J Bone Joint Surg Br* 2010, 92(1):83-91.
16. Moosmayer S, Lund G, Seljom US, Haldorsen B, Svege IC, Hennig T, Pripp AH, Smith HJ: Tendon repair compared with physiotherapy in the treatment of rotator cuff tears: a randomized controlled study in 103 cases with a five-year follow-up. *J Bone Joint Surg Am* 2014, 96(18):1504-1514.
17. Paavola M, Kanto K, Ranstam J, Malmivaara A, Inkinen J, Kalske J, Savolainen V, Sinisaari I, Taimela S, Jarvinen TL *et al*: Subacromial decompression versus diagnostic arthroscopy for shoulder impingement: a 5-year follow-up of a randomised, placebo surgery controlled clinical trial. *Br J Sports Med* 2021, 55(2):99-107.
18. Skou ST, Roos EM, Laursen MB, Rathleff MS, Arendt-Nielsen L, Simonsen O, Rasmussen S: A Randomized, Controlled Trial of Total Knee Replacement. *N Engl J Med* 2015, 373(17):1597-1606.

Appendix 10. Major guideline recommendations on subacromial decompression surgery

Guideline body/organisation	Recommendation
European Society for Surgery of the Shoulder and the Elbow	No recommendation for or against subacromial decompression surgery
British Elbow and Shoulder Society/British Orthopaedic Association 2014	Recommended in the absence of a rotator cuff tear if impingement symptoms fail to resolve with nonoperative treatment
NHS England in partnership with NHS Clinical Commissioners, the Academy of Medical Royal Colleges, NHS Improvement and the National Institute for Health and Care Excellence (Evidence-Based Interventions: Guidance for CCGs 2018)	Recommended for patients with pure subacromial shoulder impingement who have persistent or progressive symptoms, despite adequate nonoperative treatment
Dutch Orthopaedic Association 2020	Not recommended
American Academy of Orthopaedic Surgeons guidelines	No recommendation for or against subacromial decompression surgery
Australian Orthopaedic Association (Shoulder and Elbow Society of Australia) December 2017 Statement	Should only be performed for symptoms that are significant and persistent and that have not responded to non-operative care, including injections and physiotherapy
Canadian Medical Association and Canadian Orthopaedic Association-Arthroscopy Association of Canada	No recommendation for or against subacromial decompression surgery

Appendix 11. Common orthopaedic procedures, body of evidence and recommendations by clinical guidelines

Procedure	Body of evidence	Guideline recommendations
Arthroscopic anterior cruciate ligament reconstruction	Observational and limited RCT evidence suggests ACL reconstruction is not superior to non-surgical treatment	Clinical Practice Guidelines developed by the American Academy of Orthopaedic Surgeons (AAOS) recommend ACL reconstruction in active young adult (18-35) patients with an ACL tear. The strength of the recommendation was moderate. The guidelines indicate limited evidence to support non-surgical management for less active patients with less clinically assessed laxity. Furthermore, when ACL reconstruction is indicated, moderate evidence supports reconstruction within five months of injury to prevent secondary damage to the articular cartilage and menisci.
Arthroscopic meniscal repair of the knee	Evidence based on mostly observational studies suggests that meniscal repair has better outcomes than meniscectomy.	The 2018 British Association for Surgery of the Knee (BASK) Arthroscopic Meniscal Surgery Treatment Guidance was developed to provide an evidence-based national treatment guideline for patients with meniscal lesions of the knee. The Guideline group agreed to four possible treatment recommendations for meniscal lesions: (i) urgent arthroscopic meniscal surgery; (ii) consider arthroscopic meniscal repair; (iii) consider non-urgent arthroscopic partial meniscectomy; and (iv) optimized non-surgical treatment and re-assessment. Arthroscopic meniscal repair was recommended to preserve the meniscus when a reparable target lesion was identified following an acute injury. This decision was to be made by a clinician on a case-by-case basis in careful consultation with the patient. Though the guideline development process was informed by published and unpublished clinical and epidemiological evidence, the recommendations for arthroscopic meniscal repair were based on mostly indirect evidence and low-quality observational studies. The guideline group highlighted this as a priority area for further search.
Arthroscopic partial meniscectomy of the knee	Evidence based on RCT evidence suggests APM does not show clinically important benefit over non-operative treatment.	Consensus statements from specialist knee societies do not recommend APM in patients with knee pain and a meniscal tear, especially in patients with significant or end-stage osteoarthritis. It is only recommended in patients with an 'unstable' pattern of meniscal tear visible on magnetic resonance imaging that corresponds with meniscal ('mechanical') type symptoms and that it should only be performed in patients who have failed a period of non-surgical treatment.
Arthroscopic rotator cuff repair	Evidence based on RCT evidence showed no clinically important benefits of arthroscopic RCR over non-operative care.	Evidence from AAOS guideline recommendations suggests that physical therapy or operative treatment can be used for the treatment of patients with rotator cuff tears as they both result in significant improvement in patient-reported outcome measures (PROMs). Evidence demonstrates no preferential support for open or arthroscopic repairs, but the arthroscopic-only technique is associated with better short-term improvement in postoperative recovery of motion and decreased VAS scores based on individual RCTs.
Arthroscopic subacromial decompression	Evidence based on RCT evidence showed no clinically important benefits of subacromial decompression over non-operative care.	Guidelines have provided inconsistent recommendations on subacromial decompression surgery for subacromial impingement syndrome, with the majority not making a recommendation for or against the procedure. The British Elbow and Shoulder Society (BESS)/British Orthopaedic Association (BOA) guidelines recommend subacromial decompression surgery in the absence of a rotator cuff tear if impingement symptoms fail to resolve with nonoperative treatment. In a recent Rapid Recommendation published in the British Medical Journal, the guideline panel made a strong recommendation against subacromial decompression surgery in light of recent evidence, including an RCT, which showed no clinically important differences between ASAD and investigational arthroscopy or no treatment for pain and function. In updated guidance published by National Health Service (NHS) England, ASAD is recommended for patients with pure subacromial shoulder impingement who have persistent or progressive symptoms, despite adequate non-operative treatment.

Carpal tunnel decompression	Evidence based on RCT evidence showed surgical treatment relieved symptoms significantly better than non-surgical treatment.	The Commissioning Guide for the treatment of CTS developed by the Surgical Speciality Associations and Royal College of Surgeons recommend open or endoscopic decompression of CTS in secondary care for persistent severe symptoms that do not improve with splinting at night, analgesics, and corticosteroid injection for up to 12 weeks. Although no preference was given to either procedure because of the equivocal evidence, it was suggested endoscopic procedures might result in greater patient satisfaction whilst being more costly. It was recommended that open surgery be reserved for elderly patients with multiple comorbidities. Clinical Practice Guidelines developed by the AAOS strongly recommend surgical treatment of CTS compared to nonoperative treatments such as splinting, nonsteroidal anti-inflammatory drugs (NSAIDs), and a single steroid injection. There was limited evidence to support endoscopic release over open release based on possible short-term benefits.
Lumbar spine decompression	Evidence based on RCT evidence showed similar effects for decompression and non-surgical treatment.	The NICE Clinical Guideline recommends that spinal decompression should be considered for people with sciatica when non-surgical treatment has not improved pain or function and their radiological findings are consistent with sciatic symptoms.
Lumbar spine fusion	Evidence based on RCT evidence showed similar effects for LSF and non-surgical treatment.	The NICE Clinical Guideline does not recommend LSF for people with low back pain other than in the context of an RCT due to the lack of evidence of clinical effectiveness.
Total hip replacement	There are no individual RCTs that have compared THR with non-operative care, no treatment, placebo, or sham surgery for the treatment of end-stage OA.	For patients with end-stage osteoarthritis of the hip, both THR and resurfacing arthroplasty are recommended as treatment options only if the prostheses have rates/projected rates of revision of 5% or less at ten years. Guidelines from other bodies such as OARSI and European League Against Rheumatism (EULAR) recommend a hip replacement for patients with radiographic evidence of hip OA who have refractory pain and disability. The evidence for these recommendations is based on head-to-head comparisons between different types of hip prosthesis and uncontrolled studies that have used prosthesis survival as the primary outcome measure.
Total knee replacement	There are RCTs comparing TKR with no treatment, placebo, or sham surgery for the treatment of end-stage OA. One RCT compared TKR followed by non-surgical treatment versus non-surgical treatment alone (exercise, education, dietary advice, use of insoles, and analgesics) in patients with moderate-to-severe knee OA (published in 2015)	The first-line treatment for patients with hip OA is the same as for knee OA, as recommended by NICE Clinical Guideline for Osteoarthritis: care and management. For patients who experience joint symptoms (pain, stiffness, and reduced function) that have a substantial impact on their QoL and are refractory to non-surgical treatment, they recommend joint replacement surgery. Total knee replacement is the preferred surgical option in those with symptomatic OA affecting the entire tibiofemoral joint. Guidelines from other bodies such as OARSI and EULAR also recommend TKR for patients with radiographic evidence of knee OA who have refractory pain and disability. The evidence base for these recommendations is built wholly on observational retrospective studies that have often used prosthesis survival as the primary outcome measure.

ACL, anterior cruciate ligament; APM, arthroscopic partial meniscectomy ASAD, arthroscopic subacromial decompression; CTS, carpal tunnel syndrome; LSF, lumbar spine fusion; OA, osteoarthritis; RCT, randomised controlled trial; THR, total hip replacement; TKR, total knee replacement
